# State-dependent inhibition of LRRC8 volume-regulated anion channels by DCPIB

**DOI:** 10.1085/jgp.202614024

**Published:** 2026-09-22

**Authors:** Toshiki Yamada, Erkan Karakas, Jerod S. Denton

**Affiliations:** 1 Department of Anesthesiology, Vanderbilt University Medical Center, Nashville, TN, USA; 2 Department of Molecular Physiology and Biophysics, Vanderbilt University, Nashville, TN, USA; 3 Department of Pharmacology, Vanderbilt University Medical Center, Nashville, TN, USA

## Abstract

DCPIB is the prototypical inhibitor of leucine-rich repeat containing protein 8 volume-regulated anion channels (VRACs), yet the structural basis and state dependence of inhibition remain unclear. Here, we used chimeric channels, targeted mutagenesis, and electrophysiology to define the determinants of DCPIB inhibition and elucidate how drug binding is coupled to channel gating. Effective inhibition required coordinated contributions from extracellular loop 1 (EL1) and transmembrane domain 2 (TM2), which together form a functional module governing access to and stabilization of DCPIB-bound states. Within TM2, a single hydrophobic residue strongly influenced inhibition, indicating that subtle differences in helix packing shape drug sensitivity. Using the homo-heptameric 8C-8A(IL1^25^) channel as a defined model, we found that DCPIB acts exclusively from the extracellular side, inhibits cooperatively, and enhances voltage-dependent inactivation. Mutational analysis showed that DCPIB interacts permissively with R103 of the outer constriction site (OCS) but does not depend on it for inhibition, suggesting that the inhibitor intercalates between subunits within a hydrophobic cleft to achieve stable pore engagement. Charged substitutions within TM2 provided functional evidence that DCPIB penetrates beyond the OCS. Mutations at positions implicated in lipid interactions markedly altered DCPIB efficacy without affecting volume sensitivity, indicating that productive drug–channel interactions, rather than lipid gating itself, are required for inhibition. Finally, charge neutralization on the conserved N-terminal constriction enhanced DCPIB sensitivity, supporting long-range coupling within the pore. Together, these results establish DCPIB as a state-dependent VRAC inhibitor that stabilizes an inactivated channel conformation and define mechanistic principles for the rational design of more potent and selective VRAC inhibitors.

## Teaser

Yamada et al. show that the prototypical LRRC8/VRAC inhibitor DCPIB stabilizes an inactivated channel state through coordinated interactions spanning extracellular, transmembrane, and N-terminal channel domains. Their findings define key determinants of state-dependent VRAC inhibition and provide a mechanistic framework for developing more potent and selective VRAC inhibitors.

## Introduction

Volume-regulated anion channels (VRACs) are widely expressed in vertebrate cells and participate in fundamentally important physiological processes underlying cell volume homeostasis and cell signaling (Strange et al., 2019; Thone et al., 2025). VRACs are encoded by the leucine-rich repeat containing protein 8 (LRRC8) gene family, which consists of LRRC8A (8A; SWELL1), LRRC8B (8B), LRRC8C (8C), LRRC8D (8D), and LRRC8E (8E) (Qiu et al., 2014; Voss et al., 2014). Reconstitution of swelling-activated VRACs requires the expression of the essential 8A subunit along with at least one other paralog (Voss et al., 2014). Single-particle cryo-EM has revealed that LRRC8 channels are multimeric proteins containing 6 or 7 subunits (Kefauver et al., 2018; Nakamura et al., 2020; Rutz et al., 2023). Despite little conservation in their primary sequence, the three-dimensional structure of LRRC8 channels places them in the “large pore” family of ion channels, which also includes pannexins, connexins, and calcium homeostasis modulator channels (Drozdzyk et al., 2020; Michalski et al., 2020; Syrjanen et al., 2020; Syrjanen et al., 2021). Each LRRC8 subunit contains an intracellular amino-terminal (NT) domain (NTD), four transmembrane (TM) domains (TM1–4), two extracellular loops between TM1–2 and TM3–4, an intracellular loop domain between TM helix 2 (TM2)–3, and an array of leucine-rich repeat domains in the intracellular carboxyl terminus. In the assembled channel, LRRC8 subunits are separated by hydrophobic clefts of variable width that permit cell membrane phospholipids to invade and block the pore in the closed state. Lipids appear to play a direct role in channel gating because the introduction of a negatively charged aspartate group near polar lipid headgroups leads to de-lipidation of the pore and constitutive channel activation (Kern et al., 2023).

Gene-knockout studies have uncovered unexpected roles of LRRC8/VRACs in metabolic, immune, and neurological disorders that may represent new therapeutic opportunities. For example, Qiu and colleagues demonstrated that 8A/SWELL1-dependent VRACs expressed in spinal microglia release ATP in response to the inflammatory mediator sphingosine-1-phosphate in a nerve injury model of neuropathic pain. *In vivo* administration of dicumarol, a novel VRAC inhibitor identified in an FDA-approved drug library screen, alleviated mechanical allodynia in mice (Chu et al., 2023).

Jentsch and colleagues found that all five LRRC8 subunits are expressed in rodent and human islets and that deletion of 8A in pancreatic β-cells caused a delay in the glucose-induced membrane depolarization and reduced glucose-stimulated insulin secretion (GSIS) in primary cell cultures (Stuhlmann et al., 2018). Sah’s group made similar observations and additionally found that β-cell–specific ablation of 8A/SWELL1 caused impairment of GSIS and glucose tolerance *in vivo*. Furthermore, when these mice were placed on a high-fat diet, they exhibited elevated fasting glucose and worsened glucose intolerance (Kang et al., 2018). These studies raise the possibility that VRACs could be targeted with small-molecule activators to enhance GSIS in the setting of obesity.

8A/SWELL1 protein is induced by the volumetric expansion of adipocytes in models of obesity and facilitates insulin-PI3K-AKT signaling via protein–protein interactions with GRB2/Cav1 (Zhang et al., 2017). Adipocyte-specific ablation of 8A/SWELL1 reduces adiposity and impairs insulin signaling and glycemic control. Similarly, a reduction in 8A/SWELL1 expression in models of type 2 diabetes (T2D) leads to derangements of glucose homeostasis. Interestingly, however, the nonselective small-molecule VRAC inhibitor, DCPIB, improves glycemic control in T2D murine models by enhancing 8A/SWELL1 expression and insulin-PI3K-AKT signaling. While this observation supports the idea that inhibition of VRAC might offer a new therapeutic approach to improving blood glucose homeostasis in metabolic syndrome, critical validation studies will require the development of more potent and selective inhibitors. Sah and colleagues recently reported cryo-EM structure-guided medicinal chemistry efforts to identify DCPIB analogs with improved potency toward VRAC and *in vivo* efficacy (Gunasekar et al., 2022), a key step toward achieving this important goal.

Cryo-EM analysis of 8A homomeric channels in complex with DCPIB revealed that the inhibitor blocks the OCS and selectivity filter formed by a ring of positively charged arginine residues at position 103 (R103) (Kern et al., 2019). The inhibitor’s electronegative carboxylic acid group interacts electrostatically with R103, whereas its bulky hydrophobic end plugs the pore in a “cork-in-the-bottle” mechanism. Consistent with this model, mutation of R103 to phenylalanine (R103F), the corresponding residue in 8D, completely eliminates channel block. However, the R103F mutation had no effect on DCPIB inhibition in 8A-R103F/8C channels, suggesting the mechanism of inhibition is distinct in heteromers (Yamada et al., 2021).

In this study, we use chimeric and heteromeric channels, targeted mutagenesis, and electrophysiological analysis to define the structural basis of DCPIB inhibition. Our results identify extracellular loop 1 (EL1) and TM2 as key, coordinated determinants of inhibitor efficacy, with mutations in TM2 and the NTD producing the largest effects on drug sensitivity. Together with parallel changes in channel gating and DCPIB potency, these findings indicate that DCPIB inhibition is tightly coupled to channel conformational state and provide a framework for understanding how distinct structural elements contribute to state-dependent LRRC8/VRAC block.

## Materials and methods

### Drugs

DCPIB was purchased from Tocris, dissolved in DMSO (100 mM), and diluted in patch-clamp solutions immediately before use.

### Molecular biology

Site-directed mutagenesis was performed using Phusion High-Fidelity PCR Master Mix (Thermo Fisher Scientific) according to the manufacturer’s instructions. The open reading frame was fully sequenced to ensure the fidelity of mutagenesis.

### Cell lines and transfections

HCT116 cells in which all five *LRRC8* genes have been disrupted using CRISPRCas9 (HCT^*LRRC8−/−*^) were a gift from Dr. Thomas Jentsch (Leibniz-Forschungsinstitut für Molekulare Pharmakologie, Berlin, Germany). Cells were transfected using Turbofection 8.0 Transfection Reagent (OriGene Technologies) with 0.125 µg GFP (transfection marker) and 0.125–1 µg of various LRRC8 plasmids. GFP-positive cells were patch-clamped 36–48 h after transfection.

### Whole-cell patch-clamp electrophysiology

Whole-cell patch-clamp electrophysiology was performed essentially as described previously (Yamada and Strange, 2018; Yamada et al., 2021). Transfected cells were patch-clamped by using a bath solution containing (in mM): 75 CsCl, 5 MgSO_4_, 2 Ca gluconate, 12 HEPES, 8 Tris, 5 glucose, 2 glutamine, and 115 sucrose, pH 7.4 adjusted with CsOH, 300 mOsm, and a pipette solution containing (in mM): 126 CsCl, 2 MgSO_4_, 20 HEPES, 1 EGTA, 2 Na-ATP, 0.5 Na-GTP, and 10 sucrose, pH 7.2 adjusted with CsOH, 275 mOsm. A low intracellular ionic strength pipette solution was prepared by replacing 100 mM CsCl with sucrose. Cells were swollen by exposure to a hypotonic bath solution (250 mOsm) made by removal of sucrose. DCPIB inhibition experiments were performed following steady-state activation of LRRC8 currents by hypotonic cell swelling. Experiments were performed at room temperature.

A holding potential of −30 mV was used between voltage ramp and step experiments. Ramp currents were initiated by stepping the membrane potential to −100 mV for 15 ms and then ramping the voltage to +100 mV over 1 s. The test voltage was stepped back to −30 mV for 4 s before initiating the next step-ramp protocol. I–V relationships were established by stepping for 0.5 s to −80 mV followed by 2-s steps between −120 mV and +120 mV in 20 mV increments. Current over the first 5 ms following the voltage step was used for I–V relationships. Voltage-dependent inactivation (VDI) was estimated from “tail currents” recorded at −80 mV following each voltage step between −120 mV and +120 mV and normalized to that at 0 mV, where tail-current amplitude was greatest. The time constant of inactivation at the +120 mV step was estimated by fitting a single exponential to current at +120 mV for 2 s using Clampfit 10 (Molecular Devices).

### Statistical analysis

Data are presented as mean ± SEM; n represents the number of cells in patch-clamp recordings. GraphPad Prism 11 software was used for all statistical analyses. Concentration–response curves (CRCs) were generated by fitting the four-parameter Hill equation with constraints setting the bottom and top to 0 and 100, respectively. V_0.5_ was determined by fitting the Boltzmann equation. Statistical significance was determined by one-way analysis of variance (ANOVA) followed by Dunnett’s multiple comparisons test for groups >2.

### Online supplemental material

**Figure S1.**
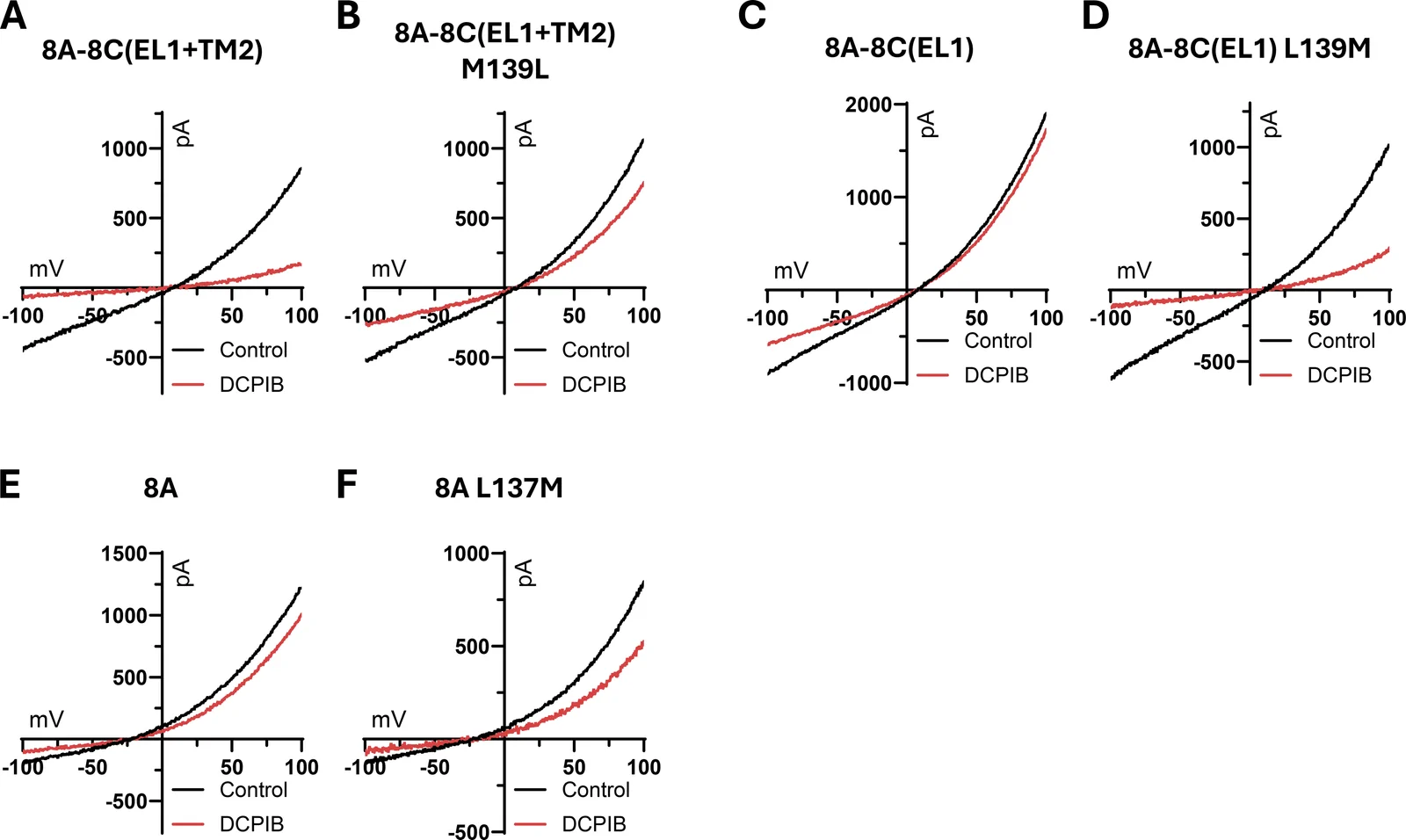
**Representative currents of the swapped point mutations in the TM2. (A–F)** Ramp currents of background chimeras and the indicated point mutants recorded from a cell dialyzed with normal (A–D) or low ionic strength (E and F) pipette solution plus cell swelling with hypotonic buffer (black), after bath application of 10 μM DCPIB (red).

**Figure S2.**
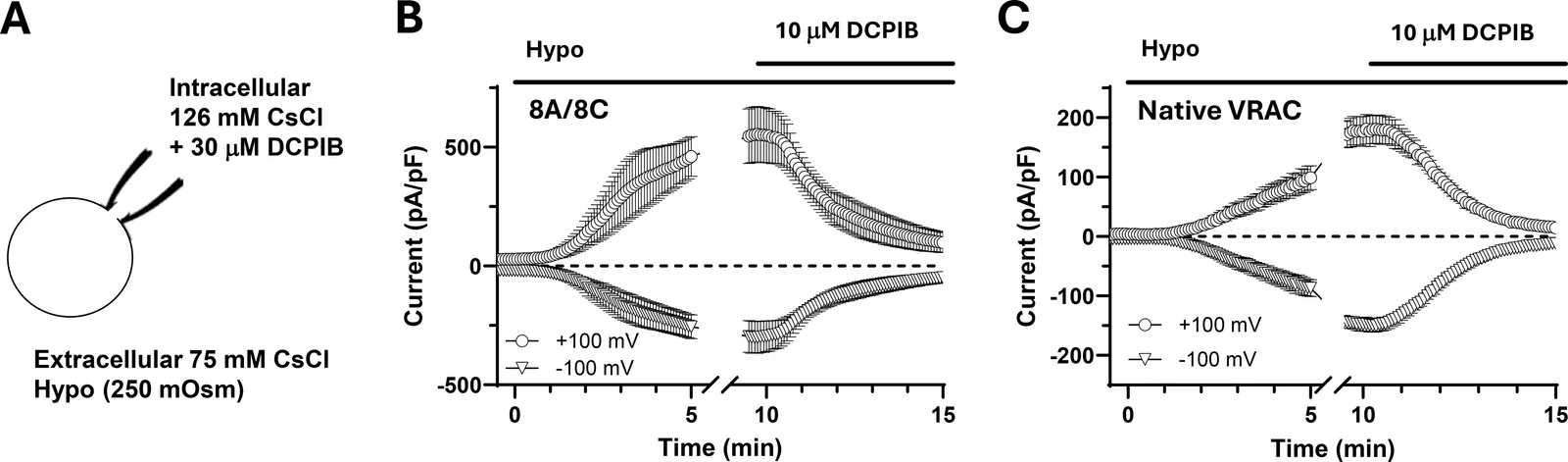
**Characteristics of DCPIB-dependent inhibition of 8A/8C heteromer and native VRAC. (A)** Intracellular dialysis with 30 µM DCPIB. **(B and C)** Time course of 8A/8C heteromer and native VRAC currents in HCT-116 cells evoked from cells dialyzed with normal pipette solution including 30 μM DCPIB and hypotonic cell swelling (8A/8C *n* = 5, native VRAC *n* = 4).

**Figure S3.**
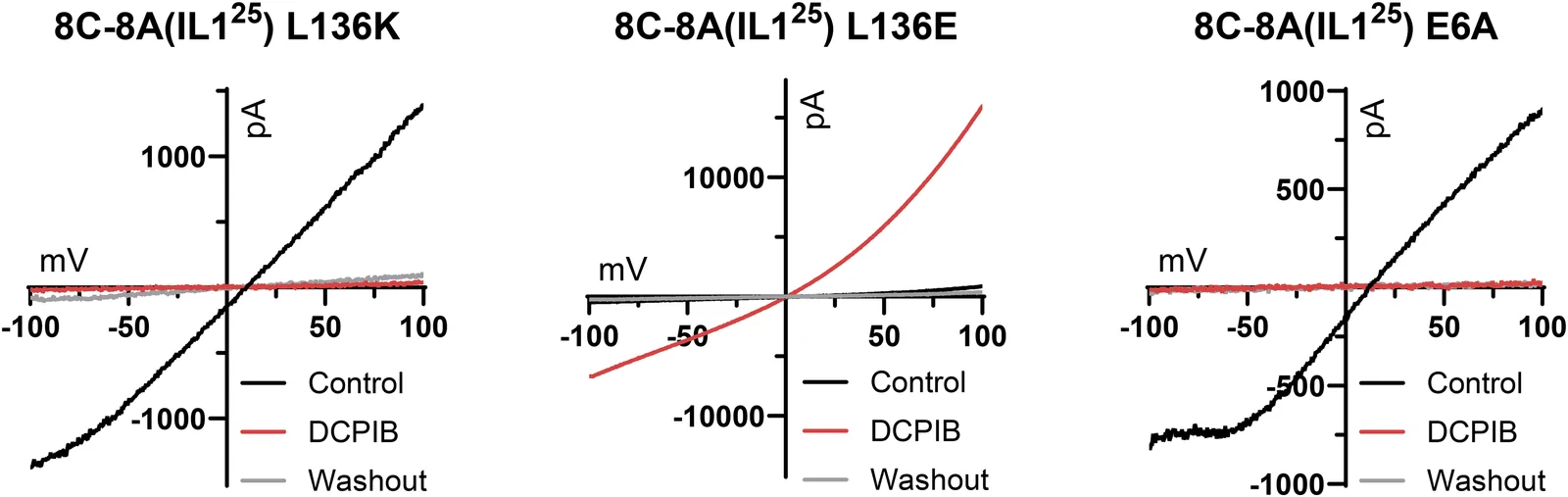
**Representative currents of drug washout in the indicated mutants.** Ramp currents of the indicated point mutants recorded from a cell dialyzed with normal pipette solution plus cell swelling with hypotonic buffer (black), after bath application of 10 μM DCPIB (red), and drug washout (gray).

**Figure S4.**
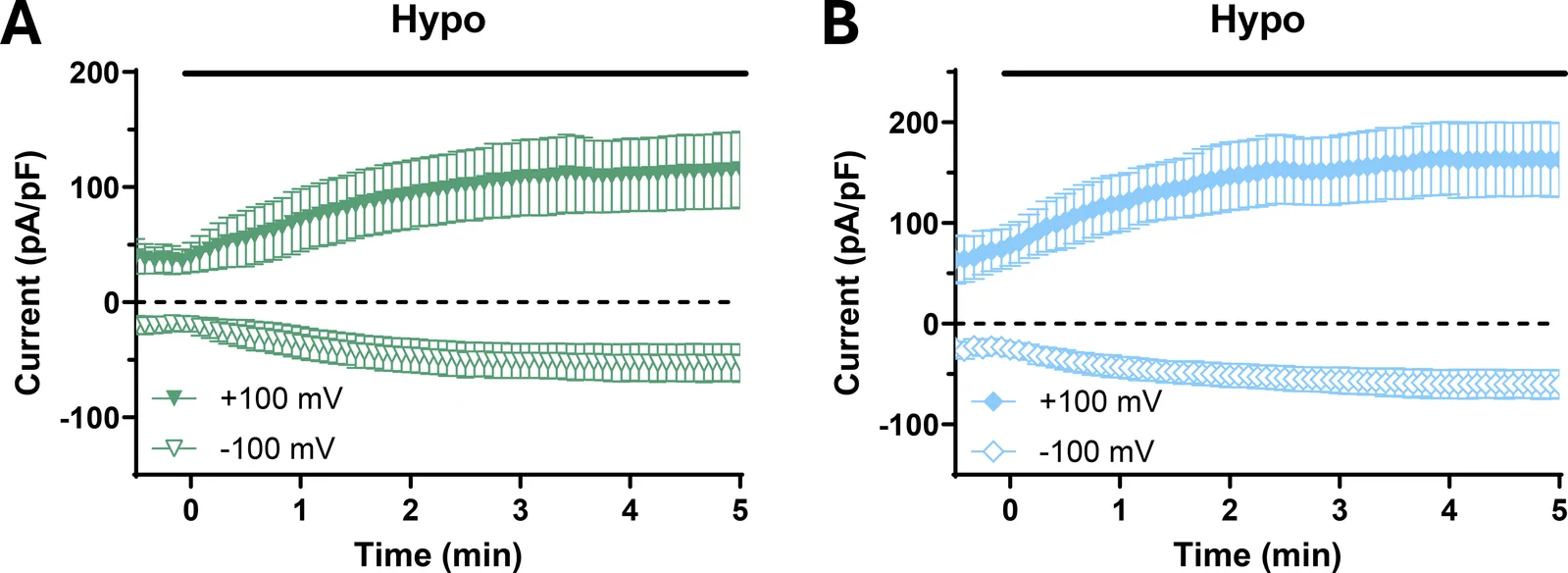
**Swelling-induced activation of 8C-8A(IL125)-M48 mutant. (A and B)** 8C-8A(IL1^25^)-M48K (A) and -M48R (B) mutants are responsive to cell swelling. Transfected cells were voltage-ramped between −100 mV and +100 mV in isotonic solution and then subjected to hypotonic cell swelling. Data are mean ± SEM (*n* = 5 each).

**Figure S5.**
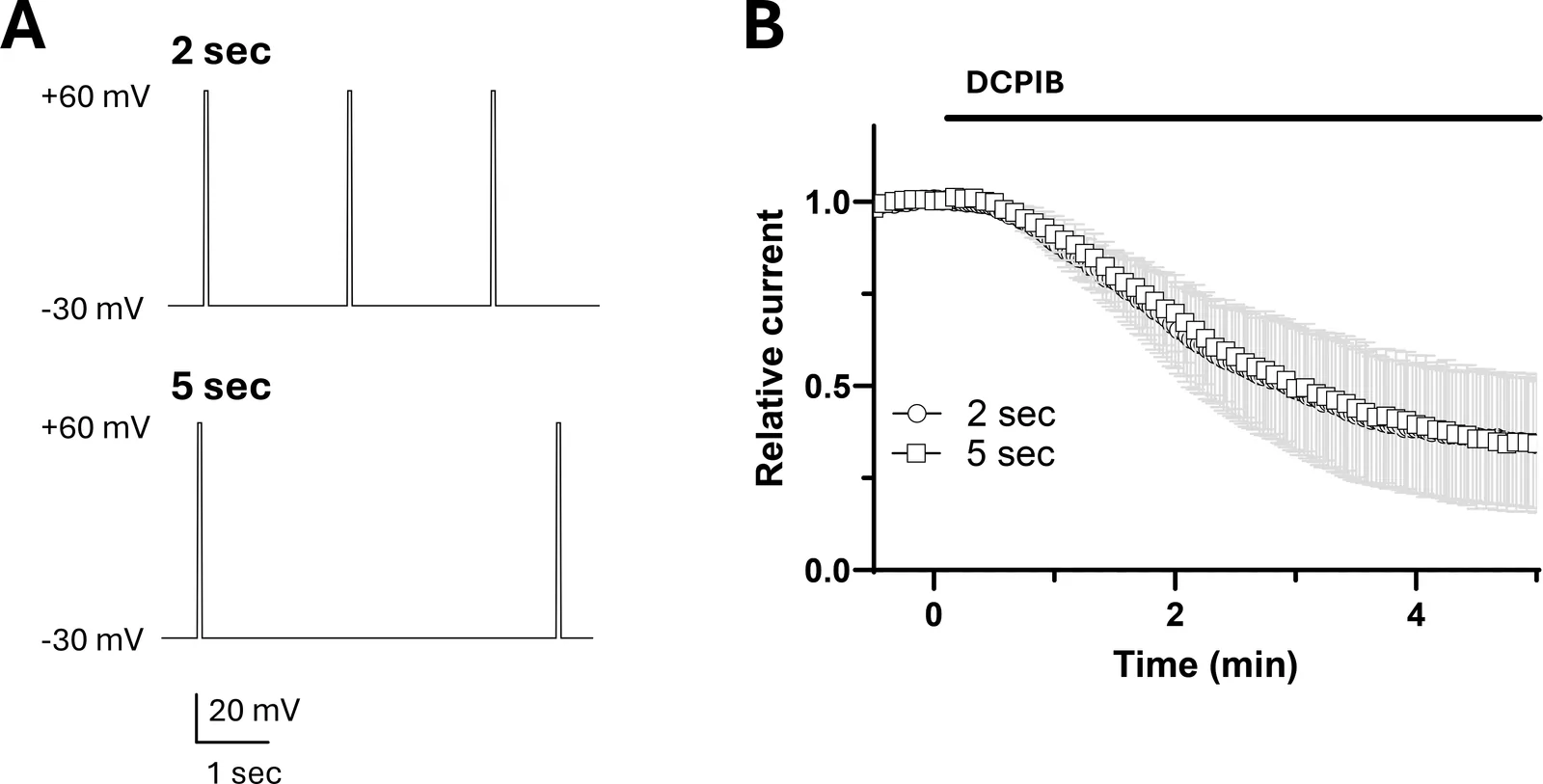
**Evaluation of use-dependent inhibition of DCPIB. (A)** Voltage-clamp protocol used to assess use-dependent block by DCPIB. **(B)** Mean ± SEM relative current evoked by a pulse (+60 mV for 50 ms) every 2 or 5 s (*n* = 3).

**Figure S6.**
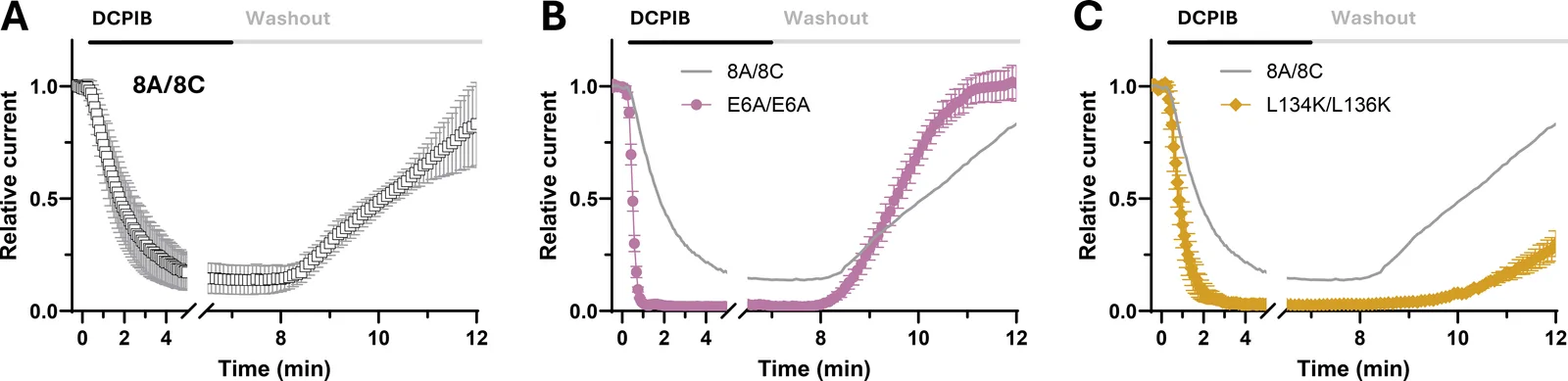
**Kinetics of washin and washout of DCPIB in heteromeric 8A/8C channels. (A–C)** Time courses of drug washout in WT (A), E6A/E6A (B), and L134K/L136K (C) LRRC8A/LRRC8C heteromers. WT data (gray line) from A is shown for comparison (8A/8C *n* = 5, E6A/E6A *n* = 5, L134K/L136K *n* = 4). Data are mean ± SEM.

Fig. S1 shows representative currents from TM2 point mutants and background chimeras under normal and low ionic strength conditions before and after DCPIB application. Fig. S2 characterizes DCPIB-dependent inhibition of 8A/8C heteromers and native VRAC currents, including intracellular DCPIB dialysis and current time courses. Fig. S3 presents representative current recordings illustrating DCPIB washout in selected mutants. Fig. S4 demonstrates that the 8C-8A(IL1^25^)-M48K and 8C-8A(IL1^25^)-M48R mutants remain responsive to hypotonic cell swelling. Fig. S5 evaluates the voltage dependence and use dependence of DCPIB inhibition using pulse protocols delivered at different frequencies. Fig. S6 shows time-course analyses of DCPIB washout in WT, glutamate 6 (E6A)/E6A, and L134K/L136K 8A/8C heteromers.

## Results

### Chimeric analysis of DCPIB-dependent LRRC8 channel inhibition

**Figure 1.**
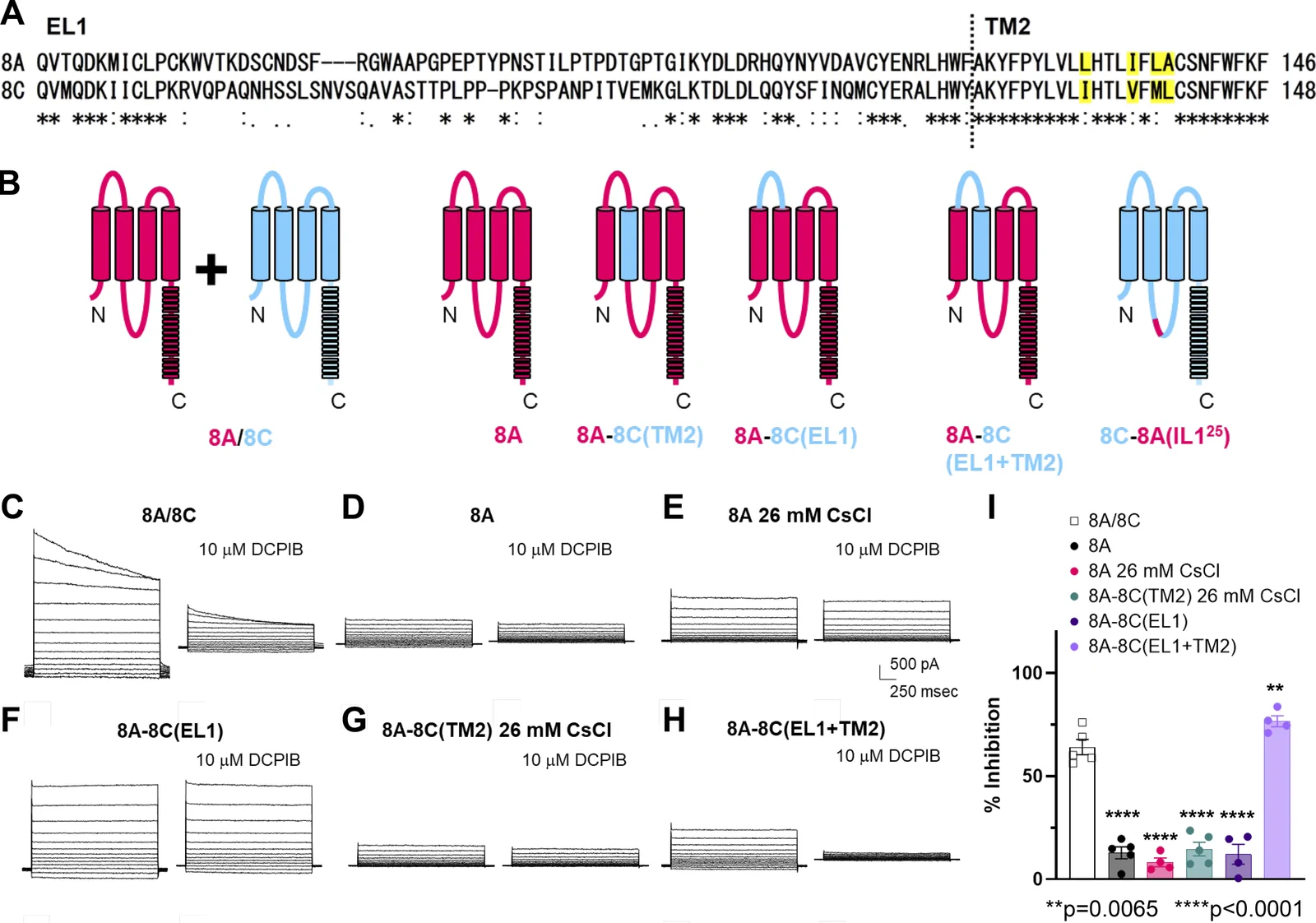
**Identification of essential regions for DCPIB inhibition in chimeras of 8A and 8C. (A)** Alignment of the first extracellular loop (EL1) and the second TM domain (TM2) of LRRC8A and LRRC8C. The differences in TM2 are highlighted in yellow. Asterisks denote identical aminio acid residues between the two aligned sequences. **(B)** Schematic images of heteromeric and chimeric LRRC8 channels. Red and blue indicate regions of 8A and 8C, respectively. **(C–H)** Representative whole-cell currents of the indicated channels recorded from a cell dialyzed with normal or low ionic strength pipette solution plus cell swelling with hypotonic buffer (left panel), after bath application of 10 μM DCPIB (right panel). The scale is the same for all traces. **(I)** Inhibition by 10 μM DCPIB at +100 mV in the indicated channels. All cells were exposed to a hypotonic buffer to activate the current. After full activation, 10 μM DCPIB was continuously applied until a new steady state was reached (3–4 min). Data are means ± SEM (8A/8C, *n* = 5; 8A, *n* = 5; 8A 26 mM CsCl, *n* = 4; 8A-8C(TM2) 26 mM CsCl, *n* = 5; 8A-8C(EL1), *n* = 4; 8A-8C(EL1+TM2), *n* = 4). **P = 0.0065; ****P < 0.0001.

We first evaluated the DCPIB sensitivity of heteromeric 8A/8C, homomeric 8A, and various chimeras containing different regions of 8A and 8C (Fig. 1, A and B). The channel constructs were expressed in HCT^*LRRC8−/−*^ cells and patch-clamped in the whole-cell configuration using CsCl-based solutions. LRRC8 currents were activated with hypotonic cell swelling before bath application of 10 µM DCPIB. Representative I–V plots are shown in Fig. 1, C–H; and Fig. S1.

Co-expression of 8A and 8C produced robust swelling-activated, outwardly rectifying, inactivating anion currents that were strongly inhibited by DCPIB (Fig. 1 C). As reported previously (Yamada et al., 2021), expression of 8A alone gives rise to small-amplitude, outwardly rectifying currents that are poorly activated by swelling and weakly inhibited by DCPIB (Fig. 1 D). Lowering intracellular ionic strength (26 mM CsCl) increased 8A current amplitude but did not improve DCPIB sensitivity (Fig. 1 E). 8A chimeras containing EL1 or TM2 from 8C, termed 8A-8C(EL1) and 8A-8C(TM2), respectively, produced volume-sensitive or low ionic strength-induced currents that were also weakly inhibited by DCPIB (Fig. 1, F and G). However, swapping both EL1 and TM2 from 8C into 8A (8A-8C(EL1+TM2)) enhanced DCPIB sensitivity by approximately fourfold (Fig. 1 H). A summary of mean ± SEM data is shown in Fig. 1 I. These results are consistent with the idea that cooperative interactions between EL1 and TM2 of 8C are needed for strong channel inhibition by DCPIB.

**Figure 2.**
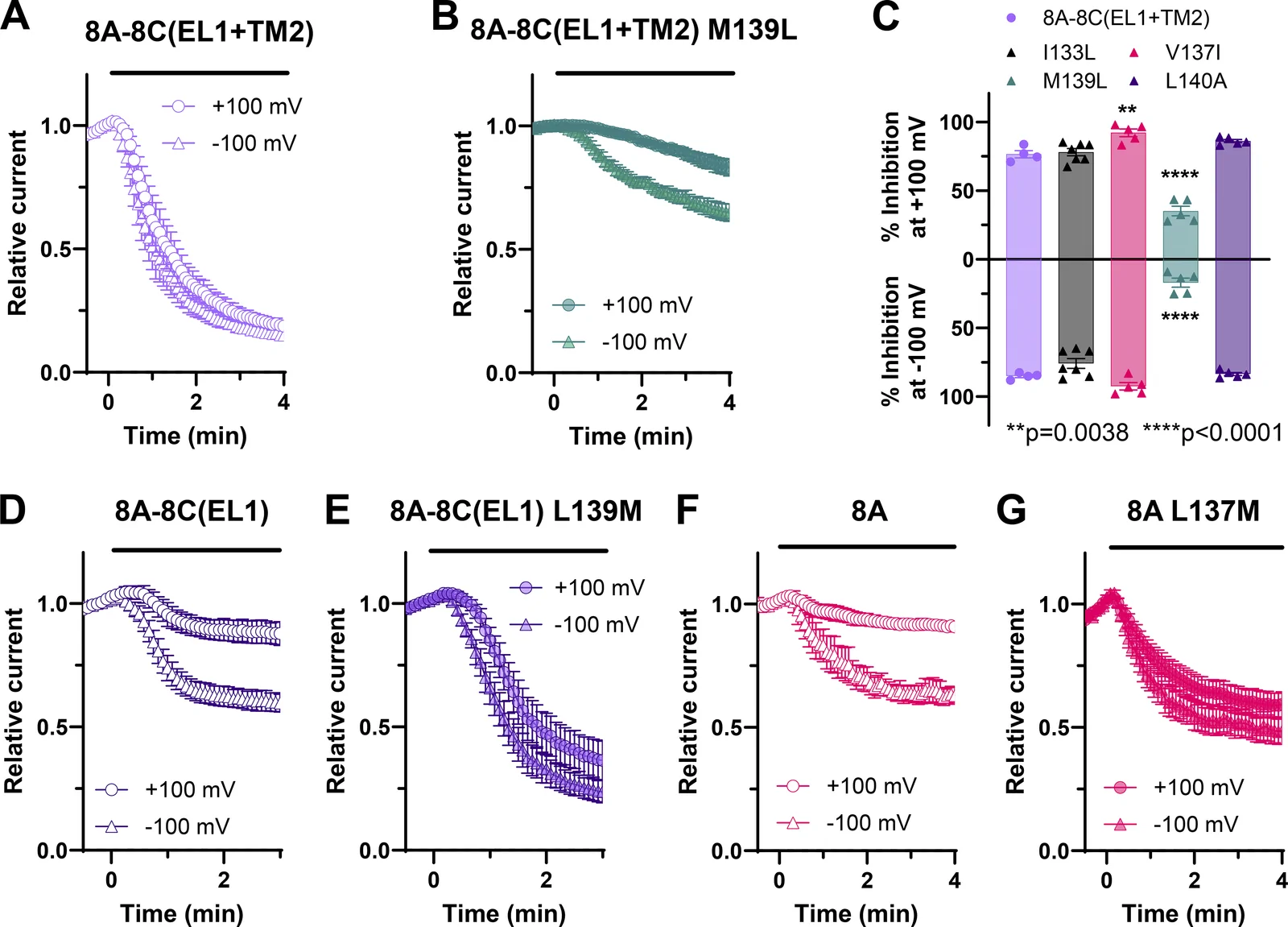
**Analysis of chimera TM2 mutations on DCPIB sensitivity. (A and B)** Time course of 10 μM DCPIB-induced inhibition of 8A-8C(EL1+TM2) (*n* = 4) and 8A-8C(EL1+TM2)-M139L (*n* = 5) currents. **(C)** Effects of single point mutations in TM2 on DCPIB inhibition (8A-8C(EL1+TM2) *n* = 4, I133L *n* = 7, V137I *n* = 5, M139L *n* = 5, L140A *n* = 5). **(D–G)** Time course of 10 μM DCPIB-induced inhibition of 8A-8C(EL1) (*n* = 4) (D and E) and 8A-8C(EL1)-L139M (*n* = 5) currents (F and G) Time course of 10 μM DCPIB-induced inhibition of 8A (*n* = 4) and 8A-L137M (*n* = 6) currents. All cells were exposed to a hypotonic buffer to activate the current. After full activation, 10 μM DCPIB was continuously applied until a new steady state was reached (3–4 min). Bars indicate the presence of 10 μM DCPIB in the bath solution. Data are means ± SEM. **P = 0.0038; ****P < 0.0001.

Sequence alignment of EL1 and TM2 of 8A and 8C reveals that while the EL1 domain is only 36% identical, TM2 is highly conserved, with only four amino acids differing between the two paralogs (Fig. 1 A, yellow highlights). The variable residues are (8A/8C numbering): L131/I133, I135/V137, L137/M139, and A138/L140. We individually swapped these residues from 8A into the 8A-8C(EL1+TM2) chimera and tested for DCPIB sensitivity. Whereas inhibition of 8A-8C(EL1+TM2) by 10 µM DCPIB was strong, voltage-independent, and reached a steady state within 4 min after bath application (Fig. 2 A), inhibition of 8A-8C(EL1+TM2)-M139L was weak, voltage-dependent, and slow (Fig. 2 B). Mutations I133L, V137I, and L140A had no significant effects on channel inhibition by DCPIB (Fig. 2 C). The unique sensitivity to mutation at the M139 position in 8C prompted us to test if the reverse mutation in 8A-8C(EL1) (i.e., L139M) would enhance DCPIB sensitivity. Indeed, this mutation converted 8A-8C(EL1) inhibition from weak and voltage-dependent (Fig. 2 D) to strong and voltage-independent (Fig. 2 E). Furthermore, the L137M mutation in 8A homomeric channels enhanced DCPIB sensitivity and eliminated voltage-dependence (Fig. 2, F and G). A summary of mean ± SEM data is shown in Fig. 2 C. Taken together, these data are consistent with the notion that hydrophobic packing and associated conformation of TM2 influence channel sensitivity to DCPIB.

### Properties of WT 8C-8A(IL1^25^) inhibition by DCPIB

**Figure 3.**
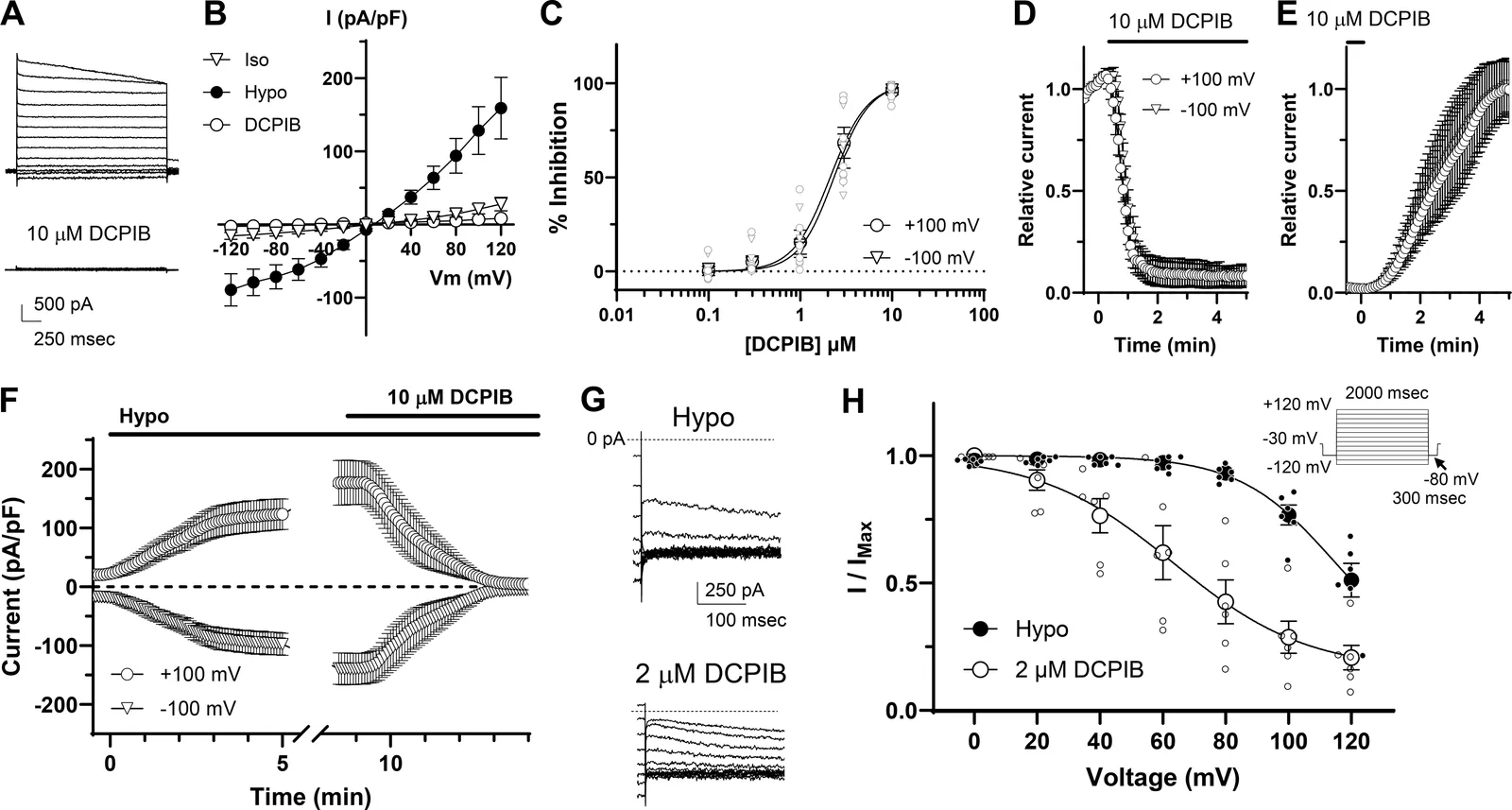
**Characteristics of DCPIB-dependent inhibition of 8C-8A(IL1** ^**25**^ **) currents. (A)** Representative whole-cell 8C-8A(IL1^25^) current recorded from a cell dialyzed with normal pipette solution and cell swelling with hypotonic buffer (upper panel), after bath application of 10 μM DCPIB (bottom panel). The scale is the same for both traces. **(B)** I–V relationships of 8C-8A(IL1^25^) from cells in isotonic, hypotonic, and hypotonic buffer with 10 μM DCPIB (*n* = 7). **(C)** CRCs of DCPIB inhibition at +100 mV and −100 mV (*n* = 6). **(D and E)** Time course of 10 μM DCPIB-induced inhibition (D, *n* = 5) and drug washout (E, *n* = 4). **(F)** Time course of 8C-8A(IL1^25^) current evoked from cells dialyzed with normal pipette solution including 30 μM DCPIB and hypotonic cell swelling (*n* = 5). All cells were exposed to a hypotonic buffer for 9 min. After full activation, 10 μM DCPIB was applied until a new steady state was reached (5 min). **(G)** Representative tail currents of 8C-8A(IL1^25^) recorded from a cell dialyzed with normal pipette solution and cell swelling with hypotonic buffer (upper panel), after bath application of 2 μM DCPIB (bottom panel). The scale is the same for both traces. **(H)** VDI of the tail currents in 8C-8A(IL1^25^) (*n* = 6). Data are mean ± SEM. The voltage-clamp protocol used to elicit tail currents is shown in the inset.

We next employed the 8C-8A(IL1^25^) chimera as a structurally defined (Takahashi et al., 2023) model channel to further probe DCPIB’s mechanism of action. 8C-8A(IL1^25^) forms homoheptameric channels with normal cell volume sensitivity, anion selectivity, outward rectification, VDI, and strong inhibition by DCPIB (Fig. 3, A and B; Takahashi et al., 2023; Yamada et al., 2021). The electrophysiological properties of 8C-8A(IL1^25^) and other channels used in this study are summarized in Table 1. Dose–response experiments revealed voltage-independent IC_50_ values of 2.1 and 2.3 μM (Fig. 3 C), and Hill coefficients of 2.15 and 2.26 at +100 mV and −100 mV, respectively (Table 2). Bath application of 10 μM DCPIB led to a complete inhibition within 2 min (Fig. 3 D) that was fully reversible upon washout (Fig. 3 E). Intracellular application of 30 μM DCPIB via the pipette solution did not prevent swelling-induced channel activation or subsequent inhibition by bath-applied DCPIB (Fig. 3 F), indicating the DCPIB binding site is accessible only from the extracellular side of the channel. This is not a unique property of 8C-8A(IL1^25^), as intracellular delivery of 30 μM DCPIB did not prevent swelling-induced activation of heteromeric 8A/8C channels or native VRAC expressed in WT HCT cells (Fig. S2). Partial channel inhibition with 2 µM DCPIB led to an ∼50-mV hyperpolarizing shift in inward tail currents recorded at −80 mV (Fig. 3, G and H). These observations suggest that DCPIB promotes a drug-induced inactivated state as part of its inhibitory mechanism of action.

**Table 1.** Electrophysiological properties of swelling-activated anion currents

Construct	Erev	Amplitude at +120 mV	Rectification	Inactivation at +120 mV	Time constant at +120 mV
	mV	pA/pF	I_+120 mV_/I_−120 mV_	I_2sec_/I_Max_	ms
**Chimera**					
8C-8A(IL1^25^)	9.9 ± 0.4 (7)	158.9± 42.2 (7)	1.80 ± 0.18 (7)	0.60 ± 0.04 (7)	2,244 ± 417 (7)
8C-8A(IL1^25^) E6A	11.9 ± 0.5 (6)	36.8 ± 3.4 (6)	1.12 ± 0.04 (6)	0.16 ± 0.02 (6)	520 ± 77 (6)
	​	​	P < 0.005	P < 0.0001	P < 0.005
8C-8A(IL1^25^) M48D	2.4 ± 0.6 (8)	81.9 ± 26.5 (8)	1.84 ± 0.11 (8)	0.83 ± 0.03 (8)	1,356 ± 257 (8)
	P < 0.0001	​	​	P < 0.005	​
8C-8A(IL1^25^) M48K	10.6 ± 0.4 (5)	145.3 ± 36.6 (5)	2.46 ± 0.10 (5)	0.69 ± 0.06 (5)	1,056 ± 90 (5)
	​	​	P < 0.005	​	​
8C-8A(IL1^25^) M48R	10.2 ± 0.5 (5)	347.6 ± 81.0 (5)	3.97 ± 0.15 (5)	0.70 ± 0.08 (5)	1,534 ± 423 (5)
	​	P < 0.005	P < 0.0001	​	​
8C-8A(IL1^25^) L105F	3.8 ± 0.5 (6)	22.1 ± 2.3 (6)	1.72 ± 0.17 (6)	0.85 ± 0.03 (6)	1,281 ± 292 (6)
	P < 0.0001	​	​	P < 0.005	​
8C-8A(IL1^25^) L105R	12.4 ± 0.7 (5)	88.0 ± 37.9 (5)	1.11 ± 0.10 (5)	0.28 ± 0.04 (5)	989 ± 120 (5)
	P < 0.05	​	P < 0.005	P < 0.0001	P < 0.05
8C-8A(IL1^25^) Q106A	10.4 ± 0.6 (5)	333.0 ± 48.4 (5)	2.19 ± 0.18 (5)	0.72 ± 0.03 (5)	1,928 ± 648 (5)
	​	P < 0.05	​	​	​
8C-8A(IL1^25^) Y108A	8.6 ± 0.6 (5)	161.5 ± 33.5 (5)	1.40 ± 0.07 (5)	0.95 ± 0.01 (5)	1,834 ± 226 (5)
	​	​	​	P < 0.0001	​
8C-8A(IL1^25^) N112A	9.2 ± 0.9 (5)	4.3 ± 0.3 (5)	1.66 ± 0.10 (5)	0.84 ± 0.06 (5)	706 ± 243 (5)
	​	P < 0.05	​	P < 0.0001	P < 0.05
8C-8A(IL1^25^) Y126F	9.3 ± 0.3 (5)	19.2 ± 5.9 (5)	0.76 ± 0.07 (5)	0.27 ± 0.05 (5)	665 ± 120 (5)
	​	​	P < 0.0001	P < 0.0001	P < 0.01
8C-8A(IL1^25^) L136E	6.3 ± 0.5 (5)	80.2 ± 25.4 (5)	2.36 ± 0.08 (5)	0.65 ± 0.09 (5)	2,634 ± 585 (5)
	P < 0.0005	​	P < 0.05	​	​
8C-8A(IL1^25^) L136K	9.5 ± 0.6 (5)	196.3 ± 54.7 (5)	1.55 ± 0.11 (5)	0.18 ± 0.01 (5)	512 ± 44 (5)
	​	​	​	P < 0.0001	P < 0.005
**Heteromer**					
8A + 8C	10.8 ± 0.3 (6)	138.3 ± 46.0 (6)	2.41 ± 0.25 (6)	0.53 ± 0.06 (6)	1,698 ± 289 (6)
8A E6A + 8C E6A	11.9 ± 0.4 (5)	96.2 ± 33.9 (5)	3.69 ± 0.60 (5)	0.56 ± 0.10 (5)	1,200 ± 202 (5)
8A T48D + 8C M48D	10.1 ± 0.7 (5)	249.7 ± 93.7 (5)	7.28 ± 0.76 (5)	0.65 ± 0.09 (5)	1,661 ± 315 (5)
	​	​	P < 0.0001	​	​
8A + 8C L105R	12.0 ± 0.5 (5)	202.0 ± 82.7 (5)	2.05 ± 0.15 (5)	0.30 ± 0.03 (5)	880 ± 223 (5)
8A Y124F + 8C Y126F	11.4 ± 0.1 (5)	102.0 ± 20.9 (5)	2.19 ± 0.08 (5)	0.31 ± 0.04 (5)	1,060 ± 130 (5)
8A L134E + 8C L136E	11.3 ± 0.4 (5)	154.5 ± 55.6 (5)	3.20 ± 0.18 (5)	0.87 ± 0.12 (5)	994 ± 298 (5)
	​	​	​	P < 0.05	​
8A L134K + 8C L136K	11.6 ± 0.5 (5)	119.0 ± 49.4 (5)	2.76 ± 0.25 (5)	0.36 ± 0.05 (5)	1,126 ± 232 (5)

Values are mean SEM. Rectification was calculated as the ratio of peak current measured at +120 mV and −120 mV. P values reflect comparisons of WT 8C-8A(IL1^25^) or 8A + 8C with mutants.

**Table 2.** Pharmacological properties of swelling-activated anion currents

Construct	IC_50_ (μM)		Hill coefficient	
	+100 mV	−100 mV	+100 mV	−100 mV
**Chimera**				
8C-8A(IL1^25^)	2.1	2.3	2.15	2.26
8C-8A(IL1^25^) E6A	0.28	0.33	1.18	1.28
	P < 0.0001	P < 0.0001	P < 0.005	P < 0.01
8C-8A(IL1^25^) M48D	412.9	109.1	0.37	0.48
	P < 0.0001	P < 0.0001	P < 0.0001	P < 0.0001
8C-8A(IL1^25^) M48K	36.9	21.7	0.65	1.53
	P < 0.0001	P < 0.0001	P < 0.0001	​
8C-8A(IL1^25^) M48R	10.2	19.4	0.75	1.33
	P < 0.0001	P < 0.0001	P < 0.0001	​
8C-8A(IL1^25^) L105F	2.1	2.4	1.91	1.87
8C-8A(IL1^25^) L105R	0.8	0.8	1.87	2.11
	P < 0.0001	P < 0.0001	​	​
8C-8A(IL1^25^) Q106A	3.1	3.5	2.28	2.48
	P < 0.0005	P < 0.0001	​	​
8C-8A(IL1^25^) Y108A	1.2	1.2	1.28	1.17
	P < 0.005	P < 0.0005	P < 0.05	P < 0.01
8C-8A(IL1^25^) N112A	5.0	5.9	2.03	2.47
	P < 0.0001	P < 0.0001	​	​
8C-8A(IL1^25^) Y126F	0.40	0.47	1.26	1.30
	P < 0.0001	P < 0.0001	P < 0.05	P < 0.05
8C-8A(IL1^25^) L136E	N.D.	N.D.	N.D.	N.D.
8C-8A(IL1^25^) L136K	0.40	0.53	1.08	1.28
	P < 0.0001	P < 0.0001	P < 0.001	P < 0.005
**Heteromer**				
8A + 8C	4.5	4.1	1.71	1.69
8A E6A + 8C E6A	0.53	0.65	1.26	1.25
	P < 0.0001	P < 0.0001	​	​
8A T48D + 8C M48D	301.3	124.9	0.68	0.59
	P < 0.0001	P < 0.0001	​	P < 0.05
8A + 8C L105R	3.3	2.59	1.58	1.49
	​	P < 0.005	​	​
8A Y124F + 8C Y126F	3.4	3.1	1.75	1.73
	​	P < 0.05	​	​
8A L134E + 8C L136E	1.9	2.2	1.24	1.34
	P < 0.0001	P < 0.0001	​	​
8A L134K + 8C L136K	1.3	1.4	1.17	1.21
	P < 0.0001	P < 0.0001	​	​

P values reflect comparisons of WT 8C-8A(IL1^25^) or 8A + 8C with mutants.

### Conceptual framework for DCPIB inhibition

Previous structural and docking studies suggest that DCPIB inhibits LRRC8 channels through a pore-blocking mechanism involving interactions at the extracellular vestibule and within inter-subunit clefts (Gunasekar et al., 2022; Kern et al., 2019). These observations led us to hypothesize a stepwise mechanism in which extracellularly applied DCPIB initially engages residues in the EL1 domain near the OCS formed by 8A-R103 or 8C-L105, then partitions into hydrophobic inter-subunit clefts (“fenestrae”), and subsequently diffuses along the membrane-facing interface toward deeper regions of the pore. In this model, the hydrophobic tail of DCPIB facilitates its stabilization within lipid-exposed clefts, enabling access to TM residues and ultimately to deeper pore-lining positions, where the drug may stabilize a blocked and/or inactivated state.

To test this model, we performed systematic mutagenesis of residues positioned along this proposed pathway, from EL1 and OCS, through inter-subunit clefts and TM interfaces, to deeper pore and NTDs, to determine how each region contributes to DCPIB binding and inhibition.

### EL1—OCS (first contact)

We first examined residues at the extracellular entry point of the pore, where DCPIB is predicted to make its initial contact. Cryo-EM and molecular docking studies have shown that DCPIB blocks the pore of homomeric 8A channels through a cork-in-bottle mechanism of action (Gunasekar et al., 2022; Kern et al., 2019). The negatively charged carboxylic acid group on DCPIB interacts electrostatically with the positively charged side chains on R103, which form the narrowest part of the conduction pore referred to here as the OCS. The bicyclic indanone ring appears to plug the pore outright and/or intercalate into the hydrophobic cleft between subunits (Gunasekar et al., 2022; Kern et al., 2019). In support of this mechanism, mutation of R103 to leucine or phenylalanine, which is present in other LRRC8 paralogs, reduces homomeric 8A inhibition by DCPIB (Yamada et al., 2021). However, R103 is not required for inhibition because 8C-8A(IL1^25^), which contains a leucine residue at the corresponding position (i.e., L105), is strongly inhibited by DCPIB (Fig. 3; Yamada et al., 2021).

**Figure 4.**
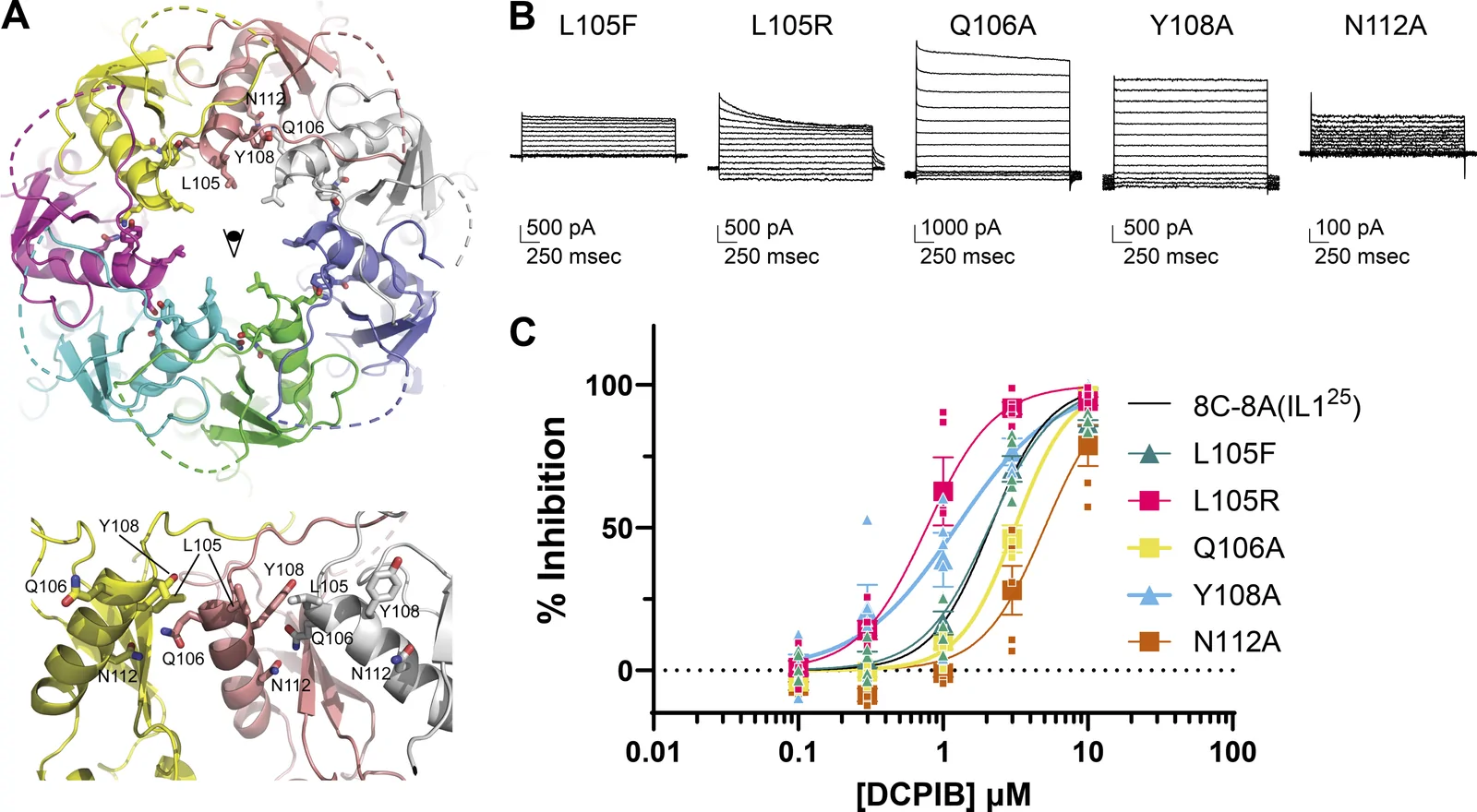
**Analysis of 8C-8A(IL1** ^**25**^ **) OCS and EL1 mutations on DCPIB sensitivity. (A and B)** Top (upper panel) and side (bottom panel) views of the extracellular domains of 8C-8A(IL1^25^) (PDB ID: 8DXN) (B) Representative whole-cell currents of the indicated mutants recorded from a cell dialyzed with normal pipette solution and cell swelling with hypotonic buffer. **(C)** CRC data recorded at +100 mV for the indicated mutants. Fit of WT CRC data (black line) from Fig. 3 C is shown for comparison. Data are mean ± SEM (8C-8A(IL1^25^) *n* = 6, mutants *n* = 5 each).

We further explored the role of the OCS in the DCPIB mechanism of action by establishing CRCs for 8C-8A(IL1^25^) channels containing at L105 an arginine residue, 8C-8A(IL1^25^)-L105R, which is found in 8A, or a phenylalanine residue, 8C-8A(IL1^25^)-L105F, which is found in 8D. Both mutants produced swelling-activated currents with variable VDI and rectification properties (Fig. 4 and Table 2). Whereas the 8C-8A(IL1^25^)-L105F mutation had no significant effect on the IC_50_ (WT 8C-8A(IL1^25^) IC_50_ = 2.1 μM vs. 8C-8A(IL1^25^)-L105F IC_50_ = 2.1 µM), the 8C-8A(IL1^25^)-L105R mutation led to an approximate threefold leftward shift in the CRC (8C-8A(IL1^25^)-L105R IC_50_ = 0.8 µM). Neither mutation affected the Hill slope of ∼2 (Table 2).

### EL1—inter-subunit cleft (entry into fenestrae)

Recent molecular docking models of a DCPIB analog (termed SN-407) bound to homomeric 8A suggest that the hydrophobic tail of DCPIB may intercalate into hydrophobic clefts between subunits near H104, Q105, and Y106 (Gunasekar et al., 2022). We therefore tested whether alanine mutations of residues occupying analogous positions in 8C-8A(IL1^25^), namely Q106A, Y108A, and N112A (Fig. 4 A), alter channel sensitivity to DCPIB. Dose–response experiments on swelling-activated currents revealed that Q106A and N112A exhibited a slight rightward shift in their DCPIB CRCs, with IC_50_s of 3.1 and 5.0 µM, respectively. In contrast, the CRC for Y108A was left-shifted with an IC_50_ of 1.2 µM (Fig. 4 C). Hill coefficients for Q106A, Y108A, and N112A were 2.28, 1.28, and 2.03, respectively (Table 2).

### TM2 mutagenesis (downward progression along lipid interface)

**Figure 5.**
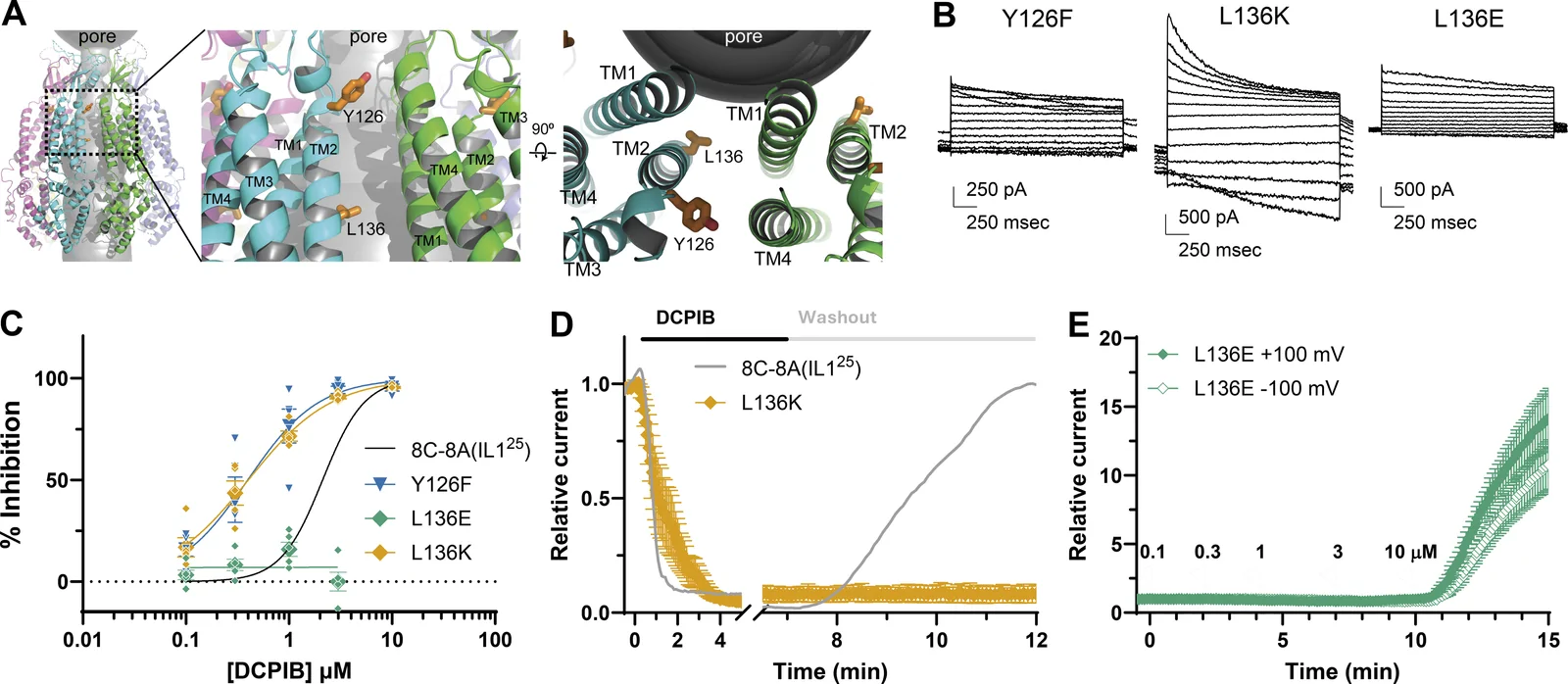
**Analysis of 8C-8A(IL1** ^**25**^ **) TM2 mutations on DCPIB sensitivity. (A)** Side (left) and top (right) views of the 8C-8A(IL1^25^) TMD (PDB ID: 8DXN) along with the solvent-accessible permeation pathway (gray surface) calculated using HOLE (Smart et al., 1996). **(B)** Representative whole-cell currents of the indicated mutants recorded from a cell dialyzed with normal pipette solution and cell swelling with hypotonic buffer. WT current is shown in Fig. 3 A. **(C)** CRC data recorded at +100 mV for the indicated mutants. Fit of WT CRC data (black line) from Fig. 3 C is shown for comparison (8C-8A(IL1^25^) *n* = 6, mutants *n* = 5 each). **(D)** Time course of drug washout in L136K. WT data (gray line) from Fig. 3 E is shown for comparison (8C-8A(IL1^25^) *n* = 4, L136K *n* = 4). After full activation, 10 μM DCPIB was applied for 7 min and then washed out for 5 min by continuous perfusion at 1 ml/min. **(E)** Time course of additional DCPIB-dependent activation of swelling-induced in L136E mutant (*n* = 5). Triangles and numbers indicate the timings and concentrations of DCPIB application, respectively. Data are the mean ± SEM.

Having identified residues in EL1 that influence DCPIB sensitivity, we next asked whether the inhibitor penetrates deeper into the channel via membrane-facing clefts. Structural data suggest that these clefts extend along the TM2-lipid interface, raising the possibility that DCPIB diffuses downward toward the pore through this pathway. In cryo-EM structures of 8C-8A(IL1^25^), lipid-like densities can be seen occupying the TM clefts and penetrating the channel wall to occlude the pore (Takahashi et al., 2023). Tyrosine 126 (Y126) is positioned near the top of TM2 (Fig. 5 A), where it likely interacts with the acyl chains and/or polar headgroups of surrounding phospholipids (Takahashi et al., 2023). Given its position at the membrane interface, Y126 may help stabilize TM2 by engaging both hydrophobic acyl chains and polar lipid headgroups. It is conceivable that externally applied DCPIB intercalating between subunits near Q106, Y108, and N112 may diffuse deeper into the cleft along the TM2-lipid interface, rendering it sensitive to mutations that destabilize these interactions. We therefore tested whether the conservative mutation Y126F, which removes the polar phenolic -OH group and hence the ability to hydrogen bond with lipid headgroups, altered DCPIB pharmacology. This mutation was shown recently to enhance sensitivity to inhibition by lipophilic drugs zafirlukast and pranlukast (Yamada et al., 2025). 8C-8A(IL1^25^)-Y126F currents were volume-sensitive and exhibited typical voltage-dependent properties (Table 1). However, the Y126F mutation led to an approximately fivefold decrease in the DCPIB IC_50_ from 2.1 µM (WT 8C-8A(IL1^25^) to 0.4 µM (8C-8A(IL1^25^)-Y126F. The Hill slope was also reduced from 2.15 in WT 8C-8A(IL1^25^) to 1.26 for 8C-8A(IL1^25^)-Y126F (Fig. 5 C and Table 2).

### Leucine 136 (transition to deeper pore engagement)

We next examined whether DCPIB interacts with deeper pore-proximal residues within TM2. Leucine 136 (L136) resides on TM2 and projects its branched hydrophobic side chain into the inter-subunit cleft (Fig. 5 A; Takahashi et al., 2023). This arrangement likely stabilizes TM2 positioning by forming hydrophobic and van der Waals contacts with surrounding acyl chains. We previously reported that mutants carrying a positively charged lysine at this position (8C-8A(IL1^25^)-L136K) exhibited enhanced sensitivity to zafirlukast and pranlukast (Yamada et al., 2025), leading us to test if this mutant also exhibits enhanced DCPIB sensitivity. L136K currents exhibited normal volume-sensitivity but abnormal voltage-dependent properties, including exaggerated VDI, loss of rectification, and time-dependent activation of inward current at hyperpolarized test potentials (Fig. 5 B and Table 1). Moreover, the IC_50_ for DCPIB-dependent inhibition of the L136K mutant was reduced approximately fourfold to 0.4 µM. The Hill slope was 1.3 (Table 2). However, unlike the WT channel (Fig. 5 D; gray trace), DCPIB-dependent inhibition was not reversed upon washout (Fig. 5 D and Fig. S3 A).

Because lysine strongly disfavors burial in the hydrophobic core, and due to the inherent rotational flexibility of TM α-helices, the L136K mutation could plausibly reorient its side chain toward the adjacent solvent-filled pore. Such reorientation could in turn enhance DCPIB inhibition by enabling favorable electrostatic interactions between the positively charged lysine ammonium group and the negatively charged carboxylate of DCPIB. To test this electrostatic model, we investigated the DCPIB sensitivity of an L136E mutant, which introduces an even more destabilizing, fully charged acidic side chain at this position. The resulting negative charge would be expected to face the pore and create electrostatic repulsion with DCPIB’s carboxylate group. 8C-8A(IL1^25^)-L136E mutant was volume-sensitive and exhibited normal voltage-dependent properties (Fig. 5 B and Table 1). Consistent with the electrostatic model, the L136E mutant exhibited no inhibition by DCPIB at doses up to 3 µM (Fig. 5 C). Unexpectedly, however, 10 µM DCPIB led to a striking additional activation of swelling-induced 8C-8A(IL1^25^)-L136E currents (Fig. 5 E and Fig. S3 B).

### “Lipid gate” mutagenesis

**Figure 6.**
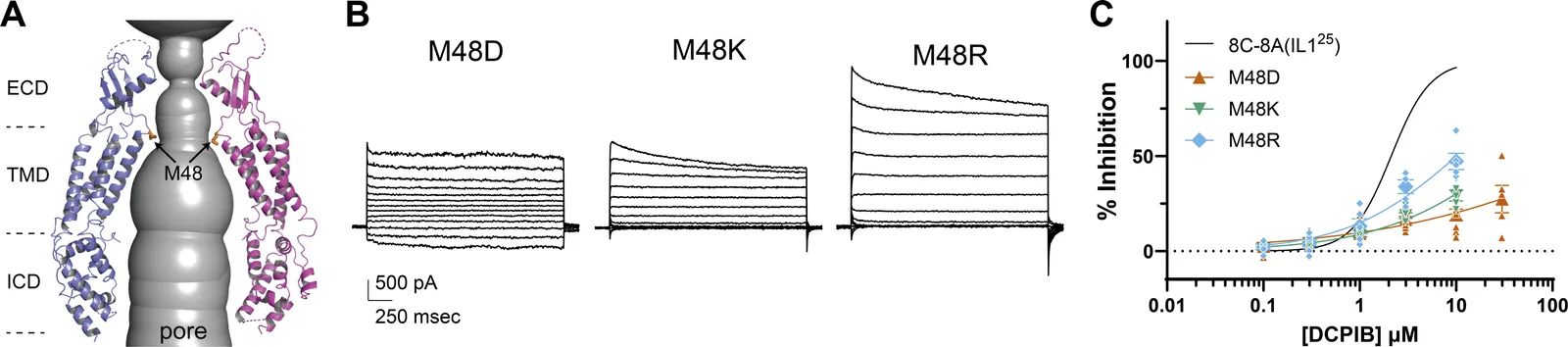
**Analysis of 8C-8A(IL1** ^**25**^ **) M48 mutations on DCPIB sensitivity. (A)** Side view of the cryo-EM structure of 8C-8A(IL1^25^) (PDB ID: 8DXN) along with the solvent-accessible permeation pathway (gray surface) calculated using HOLE (Smart et al., 1996). **(B)** Representative whole-cell currents of the indicated mutants recorded from a cell dialyzed with normal pipette solution and cell swelling with hypotonic buffer. The scale is the same for all traces. **(C)** CRC data recorded at +100 mV for the indicated mutants. Fit of WT CRC data (black line) from Fig. 3 C is shown for comparison (8C-8A(IL1^25^) *n* = 6, M48D *n* = 4–8, M48K and M48R *n* = 5 each). Data are mean ± SEM.

If DCPIB accesses the pore through lipid-facing clefts, then its inhibitory mechanism may also depend on lipid occupancy within the pore. We therefore tested whether perturbing the so-called lipid gate, comprising methionine 48 (M48) in 8C-8A(IL1^25^) (Fig. 6 A), alters DCPIB sensitivity. Cryo-EM structures of heteromeric 8A/8C (Kern et al., 2023) and 8C-8A(IL1^25^) (Takahashi et al., 2023) channels have revealed lipid densities within the central vestibule of the pore that occlude the conduction pathway. Introducing a negatively charged residue in the pore near the extracellular interface of the cell membrane is believed to electrostatically repel anionic lipid headgroups, destabilize the lipid plug, and bias the channel toward the open state (Kern et al., 2023; Takahashi et al., 2023). Membrane phospholipids potentially enter the pore through the inter-subunit clefts between TM1 and TM2. Given the important role of TM2 in DCPIB pharmacology (Fig. 5), we tested if lipid gating might play a role in channel inhibition. M48 on TM1 of 8C-8A(IL1^25^) was first mutated to aspartate (8C-8A(IL1^25^)-M48D), as this mutant is constitutively open and insensitive to cell swelling or shrinkage, consistent with defective lipid gating (Takahashi et al., 2023). Dose-response experiments revealed that 8C-8A(IL1^25^)-M48D exhibits a marked reduction in DCPIB sensitivity, with only 25% inhibition observed at a dose of 30 µM (Fig. 6 C).

We considered two possible explanations for these results. First, DCPIB could stabilize the closed state of the channel by promoting lipid occlusion of the pore. By limiting the ability of lipids to enter the pore, the M48D mutation would be expected to reduce DCPIB sensitivity. The second possibility is that the negatively charged aspartate side chain electrostatically repels the carboxylate group of DCPIB, thereby reducing inhibitor binding. We set out to distinguish between these two mechanisms by assessing the DCPIB sensitivity of mutants carrying positively charged arginine (8C-8A(IL1^25^)-M48R) or lysine (8C-8A(IL1^25^)-M48K) at this site. Both mutants exhibited significantly higher baseline current but were further activated by cell swelling (Fig. S4), consistent with at least partially intact lipid gating, and exhibited outward rectification and pronounced VDI at depolarized potentials (Fig. 6 B and Table 1). Additionally, both mutants were weakly inhibited by DCPIB. Whereas 8C-8A(IL1^25^)-M48R was inhibited by ∼50% at a concentration of 10 µM, 8C-8A(IL1^25^)-M48K was inhibited by approximately half of that at the same dose (Fig. 6 C).

### NTD mutagenesis (deepest site/endpoint)

**Figure 7.**
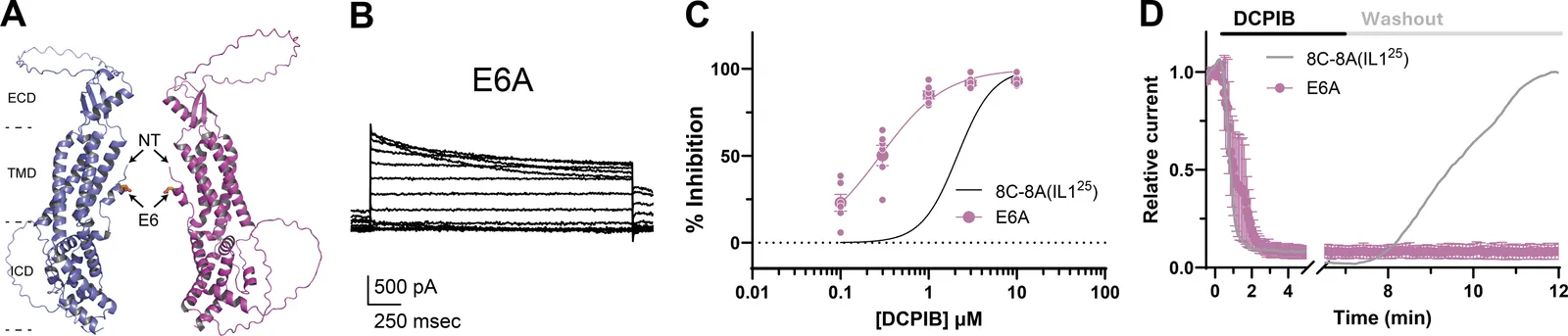
**Analysis of 8C-8A(IL1** ^**25**^ **) E6A mutation on DCPIB sensitivity. (A)** Side view of the AlphaFold3 model of the entire pore region of 8C-8A(IL1^25^) (Yamada et al., 2025). Only two opposing subunits are shown. **(B)** Representative whole-cell current of the E6A mutant recorded from a cell dialyzed with normal pipette solution and cell swelling with hypotonic buffer. **(C)** CRC data recorded at +100 mV. Fit of WT CRC data (black line) from Fig. 3 C is shown for comparison (8C-8A(IL1^25^) *n* = 6, E6A *n* = 6). **(D)** Time course of drug washout in E6A. WT data (gray line) from Fig. 3 E is shown for comparison (8C-8A(IL1^25^) *n* = 4, E6A *n* = 4). After full activation, 10 μM DCPIB was applied for 7 min and then washed out for 5 min by continuous perfusion at 1 ml/min. Data are mean ± SEM.

Finally, we asked whether DCPIB can influence regions at the deepest part of the conduction pathway by examining residues within the NTD. With the exception of 8A and 8D homomers (Liu et al., 2023; Nakamura et al., 2020), the NTD domain structure has not been resolved for any other LRRC8 channel, including 8C-8A(IL1^25^) (Takahashi et al., 2023; Yamada et al., 2021). In the 8A and 8D structures, the NTD loops back into the pore and creates a second constriction site (Liu et al., 2023; Nakamura et al., 2020). AlphaFold3 modeling and cysteine-Cd^2+^ coordination experiments suggest that, like that of 8A and 8D, the NTDs of 8C-8A(IL1^25^) extend deep into the pore and come within close proximity to one another (Yamada et al., 2021; Yamada et al., 2025; Fig. 7 A). We reported that mutation to alanine of E6A on the NTD, which is conserved across all LRRC8 paralogs, enhanced 8C-8A(IL1^25^) and 8A/8C sensitivity to inhibition by zafirlukast and pranlukast (Yamada et al., 2025). To determine if the NTD also contributes to the DCPIB mechanism of action, we assessed the sensitivity of 8C-8A(IL1^25^)-E6A in concentration–response experiments. As shown in Fig. 7 B and Table 1, E6A currents exhibited characteristic outward rectification and VDI. This mutant was approximately sevenfold more sensitive to DCPIB, with an IC_50_ of 0.28 µM. The Hill coefficient for inhibition of 8C-8A(IL1^25^)-E6A was 1.18 (Table 2). However, unlike the WT channel, but similar to 8C-8A(IL1^25^)-L136K (Fig. 5 D), DCPIB-dependent inhibition was not reversible (Fig. 7 D and Fig. S3 C).

### Mutational analysis of heteromeric 8A/8C pharmacology

**Figure 8.**
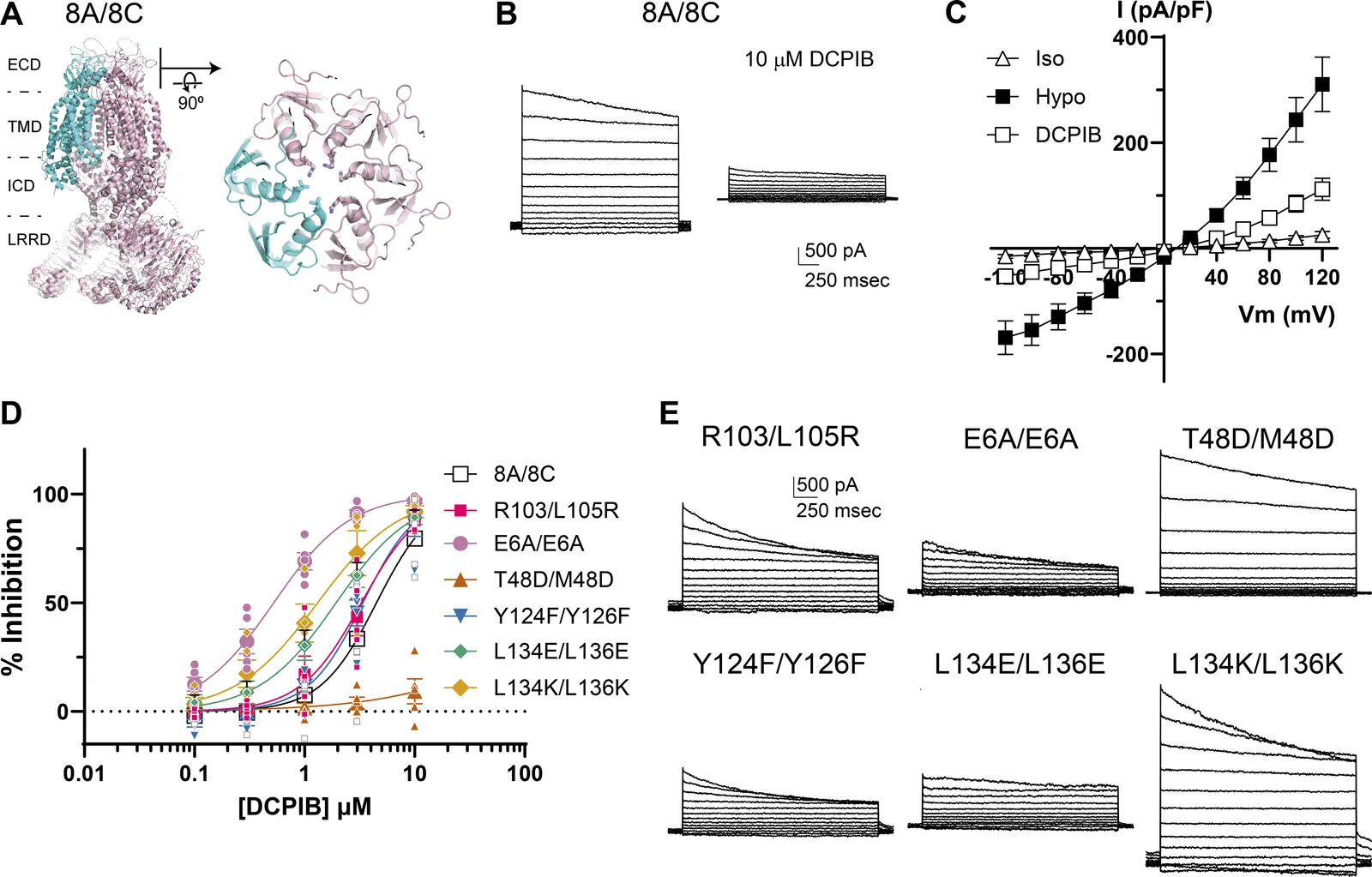
**Mutational analysis of DCPIB sensitivity in heteromeric 8A/8C currents. (A)** Side (left) and top (right) views of 8A/8C heteromer (PDB ID: 8B41) (Rutz et al., 2023). **(B)** Representative whole-cell heteromeric 8A/8C current recorded from a cell dialyzed with normal pipette solution and cell swelling with hypotonic buffer (left), after bath application of 10 μM DCPIB (right). The scale is the same for both traces. **(C)** I–V relationships of the 8A/8C heteromer from cells in isotonic, hypotonic, and hypotonic buffer with 10 μM DCPIB (*n* = 5). **(D)** CRC data recorded at +100 mV for heteromeric 8A/8C WT and the indicated mutants (8A/8C *n* = 5–6, mutants *n* = 5 each). Data are mean ± SEM. **(E)** Representative whole-cell currents of the indicated mutants recorded from a cell dialyzed with normal pipette solution and cell swelling with hypotonic buffer. The scale is the same for all traces.

8C-8A(IL1^25^) forms homoheptameric channels whereas native VRACs are heteromers containing 8A and at least one other LRRC8 paralog. Cryo-EM analyses indicate that heterologously expressed 8A/8C channels assemble as hexamers with a 4:2 stoichiometry (Lurie et al., 2025; Rutz et al., 2023; Fig. 8 A). Because 8A homomers exhibit dramatically reduced sensitivity to DCPIB (Fig. 1, D, E, and I; and Yamada et al., 2021), we tested whether select mutations that altered DCPIB sensitivity in 8C-8A(IL1^25^) also affected the pharmacology of heteromeric 8A/8C channels. As shown in Fig. 8, B and C, co-expression of 8A/8C produced swelling-activated, DCPIB-inhibitable outwardly rectifying anion currents exhibiting pronounced VDI at positive test potentials (Table 1). Concentration–response experiments showed that DCPIB inhibits 8A/8C with an IC_50_ of ∼4 µM (Fig. 8 D) and Hill coefficient of 1.7, which are similar to values observed for 8C-8A(IL1^25^) (Table 2).

In heterohexameric 8A/8C channels, the OCS is predicted to contain 4–5 charged arginine residues at position R103 and 1–2 hydrophobic leucine residues at the corresponding L105 position (Fig. 8 A). Heteromers containing WT 8A-R103 and the 8C-L105R mutation exhibited stereotypical voltage-dependent properties (Fig. 8 E and Table 1). Whereas the L105R mutation in 8C-8A(IL1^25^) led to an ∼2.5-fold increase in DCPIB sensitivity (Fig. 4 C and Table 2), introduction of arginine residues at all OCS sites in 8A/8C-L105R channels led to only a modest but significant (P < 0.05) ∼1.6-fold enhancement of DCPIB sensitivity and no change in Hill coefficient (Fig. 8 D and Table 2). Similarly, the Y124F/Y126F double mutant yielded a small yet significant (P < 0.05) reduction in DCPIB IC_50_ (Fig. 8 D and Table 2). By comparison, the E6A/E6A and L134K/L136K double mutants showed substantially larger effects, exhibiting 6–8-fold and 2.9–3.5-fold decreases in IC_50_, respectively (Fig. 8 D and Table 2). Interestingly, whereas the L136E mutation eliminated DCPIB block of 8C-8A(IL1^25^) (Fig. 5 C and Table 2), the corresponding 8A-L134E/8C-L136E mutation led to a small but significant enhancement of DCPIB sensitivity (Fig. 8 D and Table 2). Finally, the T48D/M48D double mutation completely abolished DCPIB sensitivity (Fig. 8 D and Table 2).

### Association between inactivation and DCPIB sensitivity

**Figure 9.**
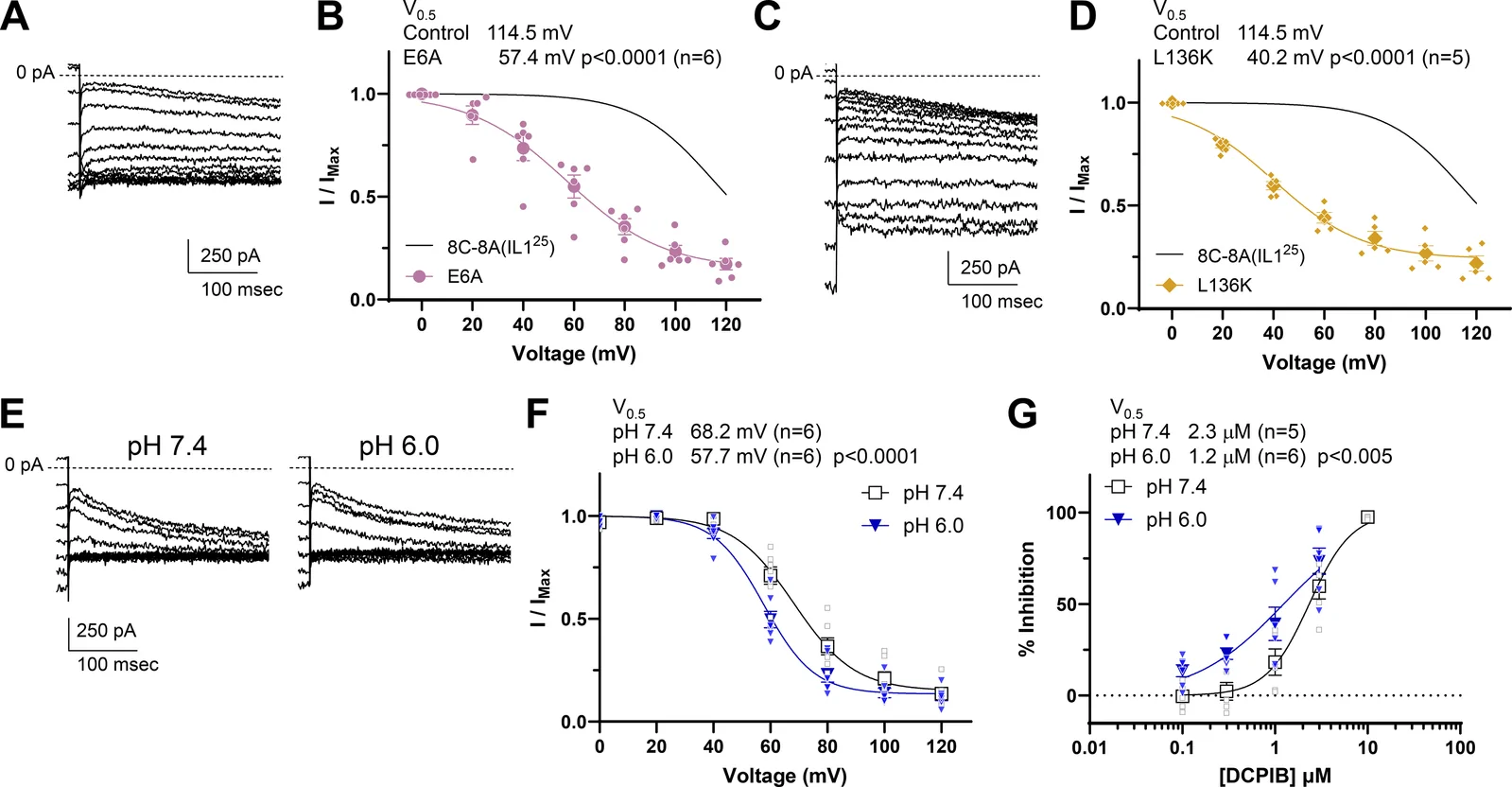
**Effects of E6A and L136K mutations and extracellular low pH on tail currents. (A)** Representative tail currents of 8C-8A(IL1^25^)-E6A mutant recorded from a cell dialyzed with normal pipette solution and swollen with hypotonic buffer. **(B)** Mean ± SEM VDI of the tail currents in 8C-8A(IL1^25^)-E6A (*n* = 5). **(C)** Representative tail currents of 8C-8A(IL1^25^)-L136K. **(D)** Mean ± SEM VDI of the tail currents in 8C-8A(IL1^25^)-L136K (*n* = 5). WT data (black line) from Fig. 3 H is shown in B and D for comparison. **(E)** Representative tail currents of native VRAC recorded from a WT HCT116 cell dialyzed with normal pipette solution and swollen with hypotonic buffer under normal (pH 7.4) and low pH (pH 6.0) bath conditions. **(F)** Mean ± SEM pH-induced enhancement of tail current inactivation (*n* = 6). After full activation and recording of the tail current at pH 7.4, the extracellular buffer was switched to a buffer at pH 6.0. **(G)** Mean ± SEM DCPIB CRC data recorded at +100 mV from HCT116 cells at pH 7.4 (open symbols, *n* = 5) and pH 6.0 (blue symbols, *n* = 6).

DCPIB enhances VDI (Fig. 3 H), suggesting that channel inhibition is functionally coupled to the inactivated state. We therefore hypothesized that mutations which enhance drug sensitivity would also shift channel gating toward the inactivated state. Consistent with this hypothesis, tail current analyses of 8C-8A(IL1^25^)-E6A and 8C-8A(IL1^25^)-L136K revealed striking 57.1 and 74.3 mV hyperpolarizing shifts in V_0.5_, respectively (Fig. 9, A–D).

We recently reported that extracellular acidification enhances both VDI and zafirlukast sensitivity of native VRAC currents in HCT cells (Yamada et al., 2025). We therefore examined whether reduced extracellular pH similarly increases sensitivity to DCPIB. As shown in Fig. 9, E and F, lowering extracellular pH from 7.4 to 6.0 produced a significant ∼10 mV hyperpolarizing shift in the tail current V_0.5_ and induced an approximately twofold reduction in DCPIB IC_50_. Taken together, these findings further support a mechanistic coupling between inactivation gating and DCPIB sensitivity.

## Discussion

**Figure 10.**
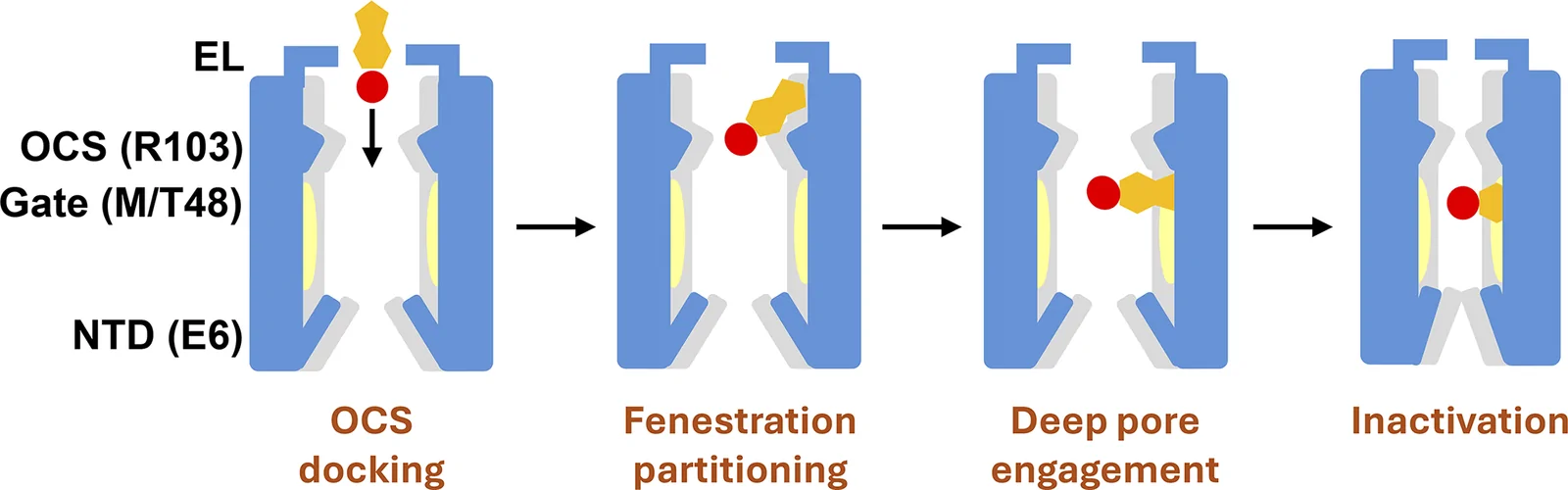
**Proposed multistep mechanism of DCPIB inhibition.** DCPIB (orange, hydrophobic moiety; red, carboxylate group) inhibits LRRC8 channels through a dynamic, state-dependent process rather than by acting as a simple static pore plug. (OCS docking) DCPIB initially binds at the OCS, where the negatively charged carboxylate can interact with residues in EL1, including R103 in LRRC8A-containing channels. Fenestration partitioning: Following initial docking, the hydrophobic portion of DCPIB partitions into membrane-facing inter-subunit fenestrations, engaging the TM1–TM2 interface. Deep pore engagement: DCPIB subsequently repositions along the pore-lining interface toward deeper regions of the channel, where inhibition is influenced by TM2 residues such as L136 and M139 and by the local electrostatic environment. Inactivation: Pore engagement stabilizes a nonconducting, inactivated channel conformation through coupling to pore geometry, lipid-associated gating mechanisms, TM2 packing, and N-terminal constriction elements.

The proposed cork-in-the-bottle mechanism for DCPIB inhibition, based on cryo-EM structures and computational docking of 8A homomers, proposes that the drug occludes the extracellular pore via interactions at the OCS near R103 while its hydrophobic tail partitions into membrane-facing fenestrations (Gunasekar et al., 2022; Kern et al., 2019). While this model provides an important structural snapshot, our results indicate that it represents an early stage of a more dynamic inhibitory process. We considered a stepwise mechanism in which DCPIB first engages EL1 residues near the outer constriction, then partitions into inter-subunit fenestrations, and subsequently progresses along membrane-facing interfaces toward deeper regions of the pore (Fig. 10). Our functional data support this sequential model but further show that effective inhibition requires coordinated interactions extending beyond the OCS. Although R103 is critical for inhibition in LRRC8A homomers (Yamada et al., 2021), consistent with an initial binding step, its dispensability in 8A/8C heteromers indicates that this interaction is not required and therefore cannot fully account for channel block, arguing against a purely static extracellular plug mechanism.

We therefore propose a dynamic, state-dependent mechanism in which DCPIB binds extracellularly but undergoes sequential repositioning within the channel, ultimately accessing deeper pore-lining regions where it stabilizes an inactivated conformation. The cooperative nature of inhibition and its enhancement of VDI support stabilization of a specific functional state rather than simple pore occlusion. In this context, the cryo-EM structure likely represents a metastable, pre-inhibitory binding pose rather than the final inhibitory configuration. Thus, while the cork-in-the-bottle model captures an important initial interaction at the outer pore, it does not account for the depth, cooperativity, and state dependence of inhibition revealed here.

Our chimeric analysis reveals that robust DCPIB inhibition requires coordinated contributions from EL1 and TM2. Individual substitution of either EL1 or TM2 from 8C into an 8A backbone was insufficient to confer strong drug sensitivity, whereas simultaneous replacement of both regions led to a pronounced increase in DCPIB efficacy (Fig. 2). This synergy indicates that EL1 and TM2 do not act independently but instead form a structural and functional module governing access to, and stabilization of, the DCPIB-bound state.

Within TM2, a single residue, M139 in 8C (corresponding to L137 in 8A), emerged as a key determinant of DCPIB sensitivity. The enhanced DCPIB sensitivity conferred by substitution of leucine with methionine at this position is potentially related to differences in side-chain flexibility and helical packing. Cryo-EM structures of homomeric 8A channels show that L137 projects into a tightly packed hydrophobic core between TM2 and TM1 (Deneka et al., 2018), where its compact, branched side chain is well suited for stabilizing α-helical interactions. In contrast, methionine possesses a longer, more flexible side chain that may weaken or loosen TM1–TM2 packing. Consistent with this idea, replacement of M139 in 8A-8C(EL1+TM2) with leucine reduced DCPIB sensitivity and restored voltage dependence (Fig. 2 B), whereas introduction of methionine at the corresponding position in DCPIB-resistant channels was sufficient to confer strong, voltage-independent inhibition (Fig. 2 E). We propose that increased local flexibility introduced by methionine destabilizes the tightly packed helical interface, thereby expanding the conformational space accessible to the pore and facilitating formation or stabilization of drug-bound states.

Experiments using the homo-heptameric 8C-8A(IL1^25^) chimera establish this construct as a robust model for probing the DCPIB mechanism of action. DCPIB inhibition was rapid, fully reversible, and voltage-independent, with Hill coefficients near 2, consistent with cooperative binding or state-dependent effects (Fig. 3, C–E; and Table 2). No evidence of use-dependent block was observed (Fig. S5). Intracellular application of DCPIB failed to inhibit channel activation, demonstrating that the binding site is accessible only from the extracellular side (Fig. 3 F and Fig. S4).

Partial DCPIB inhibition experiments revealed a large hyperpolarizing shift in tail current activation, implicating DCPIB in stabilizing a non-conducting, inactivated state (Fig. 3 H). Furthermore, enhancement of inactivation with E6A and L136K mutants, or low-pH buffer, enhances DCPIB sensitivity (Fig. 5 C, Fig. 7 C, and Fig. 9 G). These effects are inconsistent with a purely static pore occlusion model and instead support a mechanism in which DCPIB binding alters channel gating, biasing the equilibrium toward inactivation.

While cryo-EM structures of homomeric 8A channels have highlighted the importance of the OCS, formed by R103, in coordinating DCPIB binding (Gunasekar et al., 2022; Kern et al., 2019), our data show that this site is permissive rather than essential for inhibition. Substitution of the corresponding leucine in 8C-8A(IL1^25^) with arginine enhanced DCPIB potency, consistent with favorable electrostatic interactions, but phenylalanine substitution had little effect (Fig. 4 C). Moreover, heteromeric 8A/8C channels, despite containing variable numbers of arginine residues at the OCS, were effectively inhibited by DCPIB, with only modest changes in IC_50_ upon further enrichment of arginine (Fig. 8 D). These findings suggest that electrostatic interactions at the OCS can enhance binding affinity but are neither strictly required nor sufficient for high-affinity inhibition. Instead, OCS interactions likely cooperate with deeper binding determinants to stabilize the drug-bound, inactivated conformation.

Mutagenesis of residues lining the inter-subunit cleft near the extracellular vestibule revealed context-dependent effects on DCPIB sensitivity (Fig. 4 C). Alterations at Q106 and N112 modestly impaired inhibition, whereas mutation of Y108 unexpectedly enhanced DCPIB potency, suggesting that local steric or aromatic interactions within this cleft influence drug accommodation and allosteric coupling to gating.

Cryo-EM structures of heteromeric 8A/8C and 8C-8A(IL1^25^) channels reveal lipid densities within the central vestibule that occlude the conduction pathway and that are presumably displaced during channel opening (Kern et al., 2023; Lurie et al., 2025; Takahashi et al., 2023). Introducing charged residues near the extracellular membrane interface destabilizes lipid occupancy and biases channels toward an open state by electrostatically repelling anionic lipid headgroups. These observations have led to the so-called “lipid gating” hypothesis, where channel activation gating is associated with “de-lipidation” of the pore. Consistent with this model, mutation of M48 in TM1 to aspartate (M48D) yields constitutively open channels that are largely insensitive to changes in cell volume and exhibit markedly reduced sensitivity to DCPIB (Fig. 6 C), raising the possibility that intact lipid gating is required for effective channel inhibition.

To distinguish whether reduced DCPIB sensitivity in M48D reflects impaired lipid gating or local electrostatic repulsion of the negatively charged drug, we examined mutants bearing positively charged residues at this position. Notably, both M48R and M48K channels exhibited normal volume sensitivity (Fig. S4), indicating that lipid gating remains intact in these mutants. Despite preserved lipid gating, both M48R and M48K mutants displayed markedly weakened DCPIB inhibition (Fig. 6 C). This finding is consistent with a model in which the hydrophobic region of DCPIB interacts closely with the TM1–TM2 interface near M48. Introduction of positively charged side chains at this site would be expected to disrupt favorable hydrophobic interactions with the indanone core of DCPIB. Together, these results suggest that effective channel block requires productive interactions between DCPIB and a hydrophobic pocket near M48. Perturbation of this region by charged substitutions uncouples binding from inhibition, further supporting a mechanism in which DCPIB exploits lipid-associated gating transitions rather than simply occluding the pore through electrostatic interactions.

The contrasting effects of charged substitutions at L136 in 8C-8A(IL1^25^) provide functional evidence that DCPIB is influenced by electrostatic determinants located well inside the OCS. Introduction of a positively charged lysine at this position (L136K) markedly enhanced DCPIB sensitivity (Fig. 5 C), consistent with favorable electrostatic attraction between the lysine ammonium group and the negatively charged carboxylate moiety of DCPIB. In contrast, substitution with a negatively charged glutamate (L136E) abolished inhibition and instead produced drug-induced current activation (Fig. 5, C and E), as expected from electrostatic repulsion between like charges. These opposing effects suggest that DCPIB experiences the electrostatic environment of residues deep within the pore. One possibility is that DCPIB penetrates beyond the OCS and enters deeply into the TM pore or an adjacent pore-lining inter-subunit cleft, positioning its carboxylate group in close proximity to residue 136. Alternatively, the charged side chains at position 136 may influence DCPIB binding at the OCS through long-range trans-pore electrostatic coupling. Such a mechanism could arise if the electric field generated by charged residues deeper in the pore extends sufficiently to modulate interactions between DCPIB and the positively charged OCS residue R103. The different effects of L136E and L136K are also consistent with the possibility that introducing charged residues into the TM2 fenestral region alters local helix packing or TM2 orientation. Distinguishing between direct deep-pore penetration of the blocker and long-range electrostatic modulation of OCS binding will require structural and computational studies capable of resolving the position of DCPIB within the pore and quantifying electrostatic coupling between these sites.

We identified the conserved N-terminal residue E6 as a strong determinant of DCPIB sensitivity. The large leftward shift in IC_50_ observed in E6A mutants, together with enhanced inactivation and reduced Hill slopes (Fig. 7 C and Table 2), suggests that the N-terminal constriction influences higher-order cooperative or allosteric aspects of inhibition. Given that the NTD forms a second constriction site deep within the pore, we propose that DCPIB binding allosterically stabilizes NTD-mediated inactivation, further reinforcing a multi-site, state-dependent mechanism.

The differential washout behavior of DCPIB between 8C-8A(IL1^25^) and 8A/8C sensitizing mutants indicates that subunit composition governs not only apparent affinity but also the kinetics of drug dissociation. In 8C-8A(IL1^25^), mutations that enhance DCPIB block (i.e., E6A or L136K) prevent reversibility (Fig. 5 D and Fig. 7 D), consistent with stabilization of a drug-bound state with prolonged residence time. However, DCPIB readily washes out of 8A/8C containing the equivalent mutations (Fig. S6). One potential explanation is that homomeric 8C-8A(IL1^25^) assembly, derived predominantly from 8C, exhibits greater conformational flexibility, permitting induced-fit rearrangements that optimize interactions with DCPIB after binding. Such rearrangements may involve coordinated movements of pore-lining elements that effectively “tighten” around the ligand, increasing the energetic barrier for dissociation. In contrast, incorporation of 8A into heteromeric channels may impose structural constraints that favor a more pre-organized binding site, thereby limiting induced-fit stabilization and promoting more complete washout.

Additionally, the homomeric organization of 8C-8A(IL1^25^) may enable cooperative interactions among identical subunits, allowing symmetrical contributions to ligand coordination that are disrupted in the structurally heterogeneous 8A/8C assembly. This could further deepen the energy well of the DCPIB-bound state in 8C-8A(IL1^25^) relative to heteromers. An alternative, but not mutually exclusive, possibility is that binding-induced conformational changes in 8C-8A(IL1^25^) restrict solvent-accessible egress pathways, kinetically trapping DCPIB within the pore. Finally, stronger allosteric coupling between the DCPIB-binding site and channel gating in 8C-8A(IL1^25^) may stabilize a nonconducting conformation that further disfavors ligand dissociation. Together, these considerations support a model in which subunit-dependent differences in flexibility, cooperativity, and allosteric coupling shape the energy landscape of blocker binding, thereby determining both affinity and reversibility in LRRC8 channels.

The differential sensitivity of 8C-8A(IL1^25^) and 8A/8C channels carrying charged substitutions at L136 also offers instructive insights into how conformational flexibility and dynamics influence pore properties and DCPIB pharmacology. Our data suggest that the effects of substitutions at L136 cannot be explained solely by electrostatic interactions with DCPIB, but instead reflect an interplay between local charge, side-chain geometry, and channel conformational flexibility. L136 is located within TM2, where its side chain projects into a hydrophobic inter-subunit cleft rather than directly into the pore (Takahashi et al., 2023). Introduction of a positively charged lysine (L136K) enhanced DCPIB block in both the 8C-8A(IL1^25^) chimera and 8A/8C heteromers, consistent with electrostatic stabilization of the negatively charged blocker. In contrast, substitution with glutamate (L136E) abolished low-dose DCPIB inhibition in the chimera but enhanced DCPIB inhibition in 8A/8C. One explanation is that placement of a charged residue at position 136 promotes reorientation of TM2. Because glutamate is shorter and less conformationally flexible than lysine, its ability to influence pore electrostatics may depend on a larger structural rearrangement that repositions the side chain toward the pore. Such rearrangements may be favored in the more conformationally dynamic 8C-8A(IL1^25^) but constrained in 8A-containing channels. Under this model, lysine can enhance DCPIB block in both channel backgrounds because its longer, more flexible side chain can extend toward the pore without requiring substantial backbone movement, whereas glutamate exerts a strong effect only in channels that permit TM2 repacking or rotation. These findings, together with observations that destabilizing mutations at L137/M139 enhance DCPIB sensitivity, support the idea that subunit composition influences not only DCPIB sensitivity but also the structural consequences of mutations within the TM2 fenestral region. Cryo-EM and computational studies will be required to test this hypothesis.

Our findings not only clarify the molecular mechanism of DCPIB but also provide a conceptual framework for understanding how chemically distinct VRAC inhibitors preferentially target specific conformational states. These insights may inform the rational design of next-generation VRAC modulators with improved selectivity and therapeutic potential. Consistent with recent structure-guided SAR studies of DCPIB and SN-401 analogs, efficacious inhibitors appear to require a bifunctional architecture that simultaneously engages the pore and inter-subunit interfaces (Gaitan-Penas et al., 2026; Gunasekar et al., 2022). A hydrophobic anchoring moiety is needed to engage packing interactions at the TM1–TM2 interface and conserved inter-subunit clefts, whereas a terminal anionic group must penetrate deeply into the pore to establish electrostatic interactions with residues lining the selectivity filter, particularly R103 (Gaitan-Penas et al., 2026; Gunasekar et al., 2022). Importantly, increased hydrophobicity alone does not improve potency, indicating that productive inhibition depends on coordinated electrostatic and hydrophobic interactions rather than simple membrane partitioning or binding affinity (Gaitan-Penas et al., 2026). Moreover, SAR analyses indicate that the spacing between these pharmacophores is critical: shortening the distance between the hydrophobic anchor and carboxylate disrupts simultaneous engagement of the pore and interfacial binding determinants, whereas modest linker extension can enhance inhibitory potency (Gunasekar et al., 2022). Together, these observations support a model in which spatially polarized amphipathic molecules achieve high-affinity inhibition through coordinated hydrophobic anchoring and targeted pore engagement. Given that DCPIB is widely recognized as a promiscuous VRAC blocker with numerous off-target effects (Afzal et al., 2019; Figueroa and Denton, 2022), there remains a critical need for the development of more potent and selective classes of VRAC inhibitors.

A limitation of this study is that changes in DCPIB sensitivity produced by point mutations cannot be interpreted exclusively as evidence for direct drug–residue interactions. Many of the residues examined reside within tightly coupled structural modules that govern pore architecture, inter-subunit packing, lipid occupancy, and gating transitions. As a result, mutations may indirectly alter DCPIB pharmacology by perturbing channel conformation, dynamics, or state equilibria rather than by disrupting specific binding contacts. We have sought to mitigate these limitations by combining mutagenesis with structural context, bidirectional electrostatic perturbations (e.g., L136K vs. L136E), and analysis of correlated functional effects, such as changes in reversibility, Hill slope, and voltage dependence. Nevertheless, definitive assignment of direct binding interactions will require complementary approaches, including structural determination of drug-bound states and systematic chemical modification of DCPIB. Accordingly, the mechanistic model proposed here should be viewed as an experimentally constrained framework that integrates both direct and indirect contributions of multiple channel domains to DCPIB inhibition.

During revision of this manuscript, a complementary study was published that examined the mechanism of DCPIB inhibition of LRRC8 channels using electrophysiology, molecular dynamics simulations, and structure-guided DCPIB analogs (Gaitan-Penas et al., 2026). That work concluded that DCPIB remains anchored near the OCS through adaptable electrostatic interactions involving R103 and neighboring residue K51. An interesting feature of our 8C-8A(IL1^25^) chimera structure is the conformational heterogeneity observed in the D50-K51 region linking EL1β1 to TM1 (Takahashi et al., 2023). K51 adopts distinct conformations that either create or occlude an inter-subunit groove connecting the extracellular vestibule to the membrane-facing surface of the TM domain. Because the 8C-8A(IL1^25^) chimera lacks R103 yet remains highly sensitive to DCPIB, these observations provide structural support for the proposal that K51 can participate in DCPIB recognition when the canonical R103 interaction is absent. More broadly, our findings suggest that flexibility at the EL1–TM1 junction may influence accessibility and organization of the extracellular inhibitor-binding environment. Our data further indicate that although interactions at the OCS are likely important for initial DCPIB recognition, determinants of inhibitory efficacy extend beyond the OCS. The persistence of potent inhibition in channels lacking R103, the effects of mutations in TM2 and the NTD, the coupling between DCPIB sensitivity and VDI, and the state-dependent differences observed between 8C-8A(IL1^25^) and 8A/8C channels suggest that DCPIB inhibition is influenced by the broader conformational landscape of the channel. We therefore view OCS interactions as an important docking mechanism that may be coupled to additional structural rearrangements that stabilize inhibited channel states.

Alternatively, DCPIB may access its deep pore binding site through a membrane-associated route rather than by entering directly through the aqueous vestibule at the OCS. Because DCPIB is highly lipophilic, partitioning into the outer leaflet of the plasma membrane could facilitate its approach to the channel through inter-subunit gaps, with subsequent engagement of pore-lining residues responsible for inhibition. Such a mechanism is not mutually exclusive with the structural model inferred from cryo-EM studies, as electrostatic interactions involving residues near the OCS could still contribute to drug stabilization or positioning after entry. Although our findings are consistent with deep-pore inhibition by DCPIB, they do not definitively establish the route by which the inhibitor reaches its binding site, and alternative membrane-facilitated access pathways warrant future investigation.

## Supplementary Material

Review History

## Data Availability

Data will be made available from the corresponding author upon reasonable request.

## References

[bib1] Afzal, A., E.E. Figueroa, S.V. Kharade, K. Bittman, B.K. Matlock, D.K. Flaherty, and J.S. Denton. 2019. The LRRC8 volume-regulated anion channel inhibitor, DCPIB, inhibits mitochondrial respiration independently of the channel. Physiol. Rep. 7:e14303. 10.14814/phy2.14303 PMC6900491 31814333

[bib2] Chu, J., J. Yang, Y. Zhou, J. Chen, K.H. Chen, C. Zhang, H.Y. Cheng, N. Koylass, J.O. Liu, Y. Guan, and Z. Qiu. 2023. ATP-releasing SWELL1 channel in spinal microglia contributes to neuropathic pain. Sci. Adv. 9:eade9931. 10.1126/sciadv.ade9931 PMC10058245 36989353

[bib3] Deneka, D., M. Sawicka, A.K.M. Lam, C. Paulino, and R. Dutzler. 2018. Structure of a volume-regulated anion channel of the LRRC8 family. Nature. 558:254–259. 10.1038/s41586-018-0134-y 29769723

[bib4] Drozdzyk, K., M. Sawicka, M.I. Bahamonde-Santos, Z. Jonas, D. Deneka, C. Albrecht, and R. Dutzler. 2020. Cryo-EM structures and functional properties of CALHM channels of the human placenta. Elife. 9:e55853. 10.7554/eLife.55853 PMC7242029 32374262

[bib5] Figueroa, E.E., and J.S. Denton. 2022. A SWELL time to develop the molecular pharmacology of the volume-regulated anion channel (VRAC). Channels (Austin). 16:27–36. 10.1080/19336950.2022.2033511 PMC8820792 35114895

[bib6] Gaitan-Penas, H., S. Llabres, M. Pedrola, M. Gonzalez-Subias, J. Juarez-Jimenez, M. Pusch, R. Lavilla, and R. Estevez. 2026. Mechanistic insights into DCPIB inhibition of VRAC: Electrostatic control and binding plasticity. J. Gen. Physiol. 158:e202513953. 10.1085/jgp.202513953 42348849

[bib7] Gunasekar, S.K., L. Xie, A. Kumar, J. Hong, P.R. Chheda, C. Kang, D.M. Kern, C. My-Ta, J. Maurer, J. Heebink, . 2022. Small molecule SWELL1 complex induction improves glycemic control and nonalcoholic fatty liver disease in murine Type 2 diabetes. Nat. Commun. 13:784. 10.1038/s41467-022-28435-0 PMC8831520 35145074

[bib8] Kang, C., L. Xie, S.K. Gunasekar, A. Mishra, Y. Zhang, S. Pai, Y. Gao, A. Kumar, A.W. Norris, S.B. Stephens, and R. Sah. 2018. SWELL1 is a glucose sensor regulating beta-cell excitability and systemic glycaemia. Nat. Commun. 9:367. 10.1038/s41467-017-02664-0 PMC5785485 29371604

[bib9] Kefauver, J.M., K. Saotome, A.E. Dubin, J. Pallesen, C.A. Cottrell, S.M. Cahalan, Z. Qiu, G. Hong, C.S. Crowley, T. Whitwam, . 2018. Structure of the human volume regulated anion channel. Elife. 7:e38461. 10.7554/eLife.38461 PMC6086657 30095067

[bib10] Kern, D.M., J. Bleier, S. Mukherjee, J.M. Hill, A.A. Kossiakoff, E.Y. Isacoff, and S.G. Brohawn. 2023. Structural basis for assembly and lipid-mediated gating of LRRC8A:C volume-regulated anion channels. Nat. Struct. Mol. Biol. 30:841–852. 10.1038/s41594-023-00944-6 36928458

[bib11] Kern, D.M., S. Oh, R.K. Hite, and S.G. Brohawn. 2019. Cryo-EM structures of the DCPIB-inhibited volume-regulated anion channel LRRC8A in lipid nanodiscs. Elife. 8:e42636. 10.7554/eLife.42636 PMC6395065 30775971

[bib12] Liu, H., M.M. Polovitskaya, L. Yang, M. Li, H. Li, Z. Han, J. Wu, Q. Zhang, T.J. Jentsch, and J. Liao. 2023. Structural insights into anion selectivity and activation mechanism of LRRC8 volume-regulated anion channels. Cell Rep. 42:112926. 10.1016/j.celrep.2023.112926 PMC10480491 37543949

[bib13] Lurie, A., C.A. Stephens, D.M. Kern, K.M. Henn, N.R. Latorraca, and S.G. Brohawn. 2025. Assembly and lipid-gating of LRRC8A:D volume-regulated anion channels. Nat. Commun. 17:366. 10.1038/s41467-025-67052-5 PMC12795811 41388024

[bib14] Michalski, K., J.L. Syrjanen, E. Henze, J. Kumpf, H. Furukawa, and T. Kawate. 2020. The Cryo-EM structure of pannexin 1 reveals unique motifs for ion selection and inhibition. Elife. 9:e54670. 10.7554/eLife.54670 PMC7108861 32048993

[bib15] Nakamura, R., T. Numata, G. Kasuya, T. Yokoyama, T. Nishizawa, T. Kusakizako, T. Kato, T. Hagino, N. Dohmae, M. Inoue, . 2020. Cryo-EM structure of the volume-regulated anion channel LRRC8D isoform identifies features important for substrate permeation. Commun. Biol. 3:240. 10.1038/s42003-020-0951-z PMC7229184 32415200

[bib16] Qiu, Z., A.E. Dubin, J. Mathur, B. Tu, K. Reddy, L.J. Miraglia, J. Reinhardt, A.P. Orth, and A. Patapoutian. 2014. SWELL1, a plasma membrane protein, is an essential component of volume-regulated anion channel. Cell. 157:447–458. 10.1016/j.cell.2014.03.024 PMC4023864 24725410

[bib17] Rutz, S., D. Deneka, A. Dittmann, M. Sawicka, and R. Dutzler. 2023. Structure of a volume-regulated heteromeric LRRC8A/C channel. Nat. Struct. Mol. Biol. 30:52–61. 10.1038/s41594-022-00899-0 PMC9851909 36522427

[bib18] Smart, O.S., J.G. Neduvelil, X. Wang, B.A. Wallace, and M.S. Sansom. 1996. HOLE: A program for the analysis of the pore dimensions of ion channel structural models. J. Mol. Graph. 14:354–360, 376. 10.1016/s0263-7855(97)00009-x 9195488

[bib19] Strange, K., T. Yamada, and J.S. Denton. 2019. A 30-year journey from volume-regulated anion currents to molecular structure of the LRRC8 channel. J. Gen. Physiol. 151:100–117. 10.1085/jgp.201812138 PMC6363415 30651298

[bib20] Stuhlmann, T., R. Planells-Cases, and T.J. Jentsch. 2018. LRRC8/VRAC anion channels enhance beta-cell glucose sensing and insulin secretion. Nat. Commun. 9:1974. 10.1038/s41467-018-04353-y PMC5958052 29773801

[bib21] Syrjanen, J.L., K. Michalski, T.H. Chou, T. Grant, S. Rao, N. Simorowski, S.J. Tucker, N. Grigorieff, and H. Furukawa. 2020. Structure and assembly of calcium homeostasis modulator proteins. Nat. Struct. Mol. Biol. 27:150–159. 10.1038/s41594-019-0369-9 PMC7015811 31988524

[bib22] Syrjanen, J., K. Michalski, T. Kawate, and H. Furukawa. 2021. On the molecular nature of large-pore channels. J. Mol. Biol. 433:166994. 10.1016/j.jmb.2021.166994 PMC8409005 33865869

[bib23] Takahashi, H., T. Yamada, J.S. Denton, K. Strange, and E. Karakas. 2023. Cryo-EM structures of an LRRC8 chimera with native functional properties reveal heptameric assembly. Elife. 12:e82431. 10.7554/eLife.82431 PMC10049205 36897307

[bib24] Thone, F.M.B., M.M. Polovitskaya, and T.J. Jentsch. 2025. LRRC8/VRAC chloride and metabolite channels in signaling and volume regulation. Trends Biochem. Sci. 50:873–891. 10.1016/j.tibs.2025.07.001 40769835

[bib25] Voss, F.K., F. Ullrich, J. Munch, K. Lazarow, D. Lutter, N. Mah, M.A. Andrade-Navarro, J.P. von Kries, T. Stauber, and T.J. Jentsch. 2014. Identification of LRRC8 heteromers as an essential component of the volume-regulated anion channel VRAC. Science. 344:634–638. 10.1126/science.1252826 24790029

[bib26] Yamada, T., P. Bisignano, E. Karakas, and J.S. Denton. 2025. A conserved mechanism of LRRC8 channel inhibition by two structurally distinct drugs. Commun. Biol. 8:1432. 10.1038/s42003-025-08795-1 PMC12501385 41053296

[bib27] Yamada, T., E.E. Figueroa, J.S. Denton, and K. Strange. 2021. LRRC8A homohexameric channels poorly recapitulate VRAC regulation and pharmacology. Am. J. Physiol. Cell Physiol. 320:C293–C303. 10.1152/ajpcell.00454.2020 PMC8294627 33356947

[bib28] Yamada, T., and K. Strange. 2018. Intracellular and extracellular loops of LRRC8 are essential for volume-regulated anion channel function. J. Gen. Physiol. 150:1003–1015. 10.1085/jgp.201812016 PMC6028502 29853476

[bib29] Zhang, Y., L. Xie, S.K. Gunasekar, D. Tong, A. Mishra, W.J. Gibson, C. Wang, T. Fidler, B. Marthaler, A. Klingelhutz, . 2017. SWELL1 is a regulator of adipocyte size, insulin signalling and glucose homeostasis. Nat. Cell Biol. 19:504–517. 10.1038/ncb3514 PMC5415409 28436964

