# Supplementary material for: State-dependent inhibition of LRRC8 volume-regulated anion channels by DCPIB
Source: J Gen Physiol. 2026 Sep 22;158(6):e202614024. doi: 10.1085/jgp.202614024 (PMC13596092; doi:10.1085/jgp.202614024)
Supplement: Supplementary file 1 [file jgp_202614024_review_history.pdf]

State-Dependent Inhibition of LRRC8 Volume-Regulated Anion Channels by DCPIB

Toshiki Yamada, Erkan Karakas, and Jerod Denton

Corresponding Author(s): Jerod Denton, Vanderbilt University Medical Center

Review Timeline:

Submission Date:	April 7, 2026
Editorial Decision:	May 21, 2026
Revision Received:	July 20, 2026
Editorial Decision:	August 18, 2026
Revision Received:	August 21, 2026

Editor: Joseph Mindell

Transaction Report:

(Note: With the exception of the correction of typographical or spelling errors that could be a source of ambiguity, letters and reports are not edited. The original formatting of letters and referee reports may not be reflected in this compilation.)

DOI: https://doi.org/10.1085/jgp.202614024

Dr. Jerod Denton
Vanderbilt University Medical Center
Anesthesiology
T4208 MCN
1161 21st Avenue South
Nashville, TN 37221

Re: 202614024

Dear Dr. Denton,

Thank you for submitting your manuscript, entitled "State-dependent deep-pore inhibition of LRRC8 (VRAC) channels by DCPIB" to JGP. Your manuscript has now been seen by 3 reviewers, whose comments are appended below. You will see that the reviewers appreciated the significants and quality of the work, but they have raised several concerns that must be addressed prior to further consideration of the manuscript at JGP. In particular, the editors consider reviewer #1's point about expression level and several reviewers' comments about organization and streamlinig as essential to address. In this editor's reading the early results on chimeras were clearly presented, but the later results with pore mutations were difficult to track and organize. Also JGP requires the use of appropriate statistical tests to establish the significance of your interventions, particularly for the data in Figures 1 and 2 but also in the dose response data in later figures.

We would be pleased to receive a suitably revised manuscript that addresses these concerns, which will be re-reviewed, most likely by some or all of the original referees. In addition, please do not hesitate to contact me (via the editorial office) if you feel that a discussion of the reviewers' and editors' comments would be helpful.

Please submit your revised manuscript via the link below along with a point-by-point letter that details your responses to the editor's summary and reviewers' comments, as well as a copy of the text with alterations highlighted (boldfaced or underlined). If the article is eventually accepted, it would include a 'revised date' as well as submitted and accepted dates. If we do not receive the revised manuscript within one year, we will regard the article as having been withdrawn. We would be willing to receive a revision of the manuscript at a later time, but the manuscript will then be treated as a new submission, with a new manuscript number.

Please pay particular attention to recent changes to our instructions to authors in the following sections: Data presentation, Blinding and randomization and Statistical analysis, under Materials and Methods, as shown here: https://rupress.org/jgp/pages/submission-guidelines#prepare. Re-review will be contingent on inclusion of the required information (including for data added during revision) and demonstration of the experimental reproducibility of the results. Also, to improve the reproducibility of published content, we have partnered with SciScore. Authors are prompted in eJP to copy and paste the Materials and Methods section of their manuscript for a SciScore assessment when submitting their revised manuscript. Authors are encouraged (not required) to further revise their Materials and Methods if the SciScore is below 4. More information can be found here: https://rupress.org/jgp/pages/submission-guidelines#sciscore

Please note, JGP requires authors to submit Source Data used to generate figures containing gels and Western blots with all revised manuscripts (when applicable). This Source Data consists of fully uncropped and unprocessed images for each gel/blot displayed in the main and supplemental figures. If your paper includes cropped gel and/or blot images, please be sure to provide one Source Data file for each figure that contains gels and/or blots along with your revised manuscript files. File names for Source Data figures should be alphanumeric without any spaces or special characters (i.e., SourceDataF#, where F# refers to the associated main figure number or SourceDataFS# for those associated with Supplementary figures). The lanes of the gels/blots should be labeled as they are in the associated figure, the place where cropping was applied should be marked (with a box), and molecular weight/size standards should be labeled wherever possible.
Source Data files will be made available to reviewers during evaluation of revised manuscripts and, if your paper is eventually published in JGP, the files will be directly linked to specific figures in the published article.

Source Data Figures should be provided as individual PDF files (one file per figure). Authors should endeavor to retain a minimum resolution of 300 dpi or pixels per inch. Please review our instructions for export from Photoshop, Illustrator, and PowerPoint here: https://rupress.org/jgp/pages/submission-guidelines#revised

When revising your manuscript, please be sure it is a double-spaced MS Word file and that it includes editable tables, if appropriate.

Please submit your revised manuscript via this link:
Link Not Available

Thank you for the opportunity to consider your manuscript.

Sincerely,

Joseph A. Mindell, M.D., Ph.D.
On behalf of Journal of General Physiology

Journal of General Physiology's mission is to publish mechanistic and quantitative molecular and cellular physiology of the highest quality; to provide a best-in-class author experience; and to nurture future generations of independent researchers.

Reviewer #1 (Comments to the Authors):

In the manuscript titled "State-dependent deep-pore inhibition of LRRC8 (VRAC) channels by DCPIB", Yamada, Karakas & Denton propose a novel mechanism of VRAC inhibition by the small molecule DCPIB, the "best-in-class inhibitor of LRRC8/VRACs. The current structural model of VRAC inhibition by DCPIB suggests a cork-in-bottle mechanism that involves the interaction of the carboxylic group of DCPIB with the arginine residue 103 of LRRC8A homomeric channel (R103) and pore occlusion by the hydrophobic part of the small molecule (PMID: 30775971). In the current study, the authors challenge that model and propose that the mechanism of VRAC inhibition by DCPIB involves stabilization of an inactivated, possibly lipid-occluded, channel state, with contributions from TM2 packing, inter-subunit cleft geometry, lipid interactions, and the N-terminal constriction. Combining chimeric channels, targeted mutagenesis, and electrophysiological analysis, the authors aim to define the determinants of DCPIB inhibition and to elucidate how drug binding is coupled to channel functional state. The manuscript attempts to address a major, critical, and controversial aspect of VRACs: their mechanism of channel inhibition by the most commonly used inhibitor, DCPIB. Although the findings are interesting and relevant, the authors require additional experimental evidence to substantiate the proposed mechanism. Please see below my comments.

Major concerns:

1. One of my main concerns is the assumption that chimeric and mutant channel expression at the cell surface is comparable. To avoid this conflict, the authors do well in normalizing their current densities to each chimera/mutant. Although this approach may be acceptable for some questions, in experiments involving a channel with tightly regulated membrane expression, verifying membrane channel expression is critical. That will strengthen their findings. Have the authors verified channel expression and whether cells that are patched have similar expression patterns? The fact that they were co-transfected with GFP and they are fluorescently green doesn't mean they have similar channel expression at the cell surface. The representative current-voltage relationships observed in many experiments appear to vary across chimeras, and this may reflect differences in membrane channel expression or changes in their functional properties. This is evident when testing LRRC8C chimeras (L105F, Q106A, N112A, M48D. None of these mutants have properties of LRRC8C-containing VRACs, characterized by slow inactivation at positive membrane potential. In some Figures, the scale between current vs. time is different, making these comparisons hard to assess (e.g., Fig. 4B, 5B), and there is no explanation for these phenotypes beyond the fact that they occur. I will encourage the authors to present representative immunofluorescence images of fluorescent-tagged chimeras and mutants to ensure proper cell-surface expression (which will reflect proper channel folding, assembly, maturation, and trafficking).

2. I'm intrigued by the author's findings that a 30 uM application of DCPIB in the pipette solution is insufficient to prevent swelling-induced channel activation (Fig 3F), which led them to conclude that the DCPIB binding site is accessible only from the extracellular side of the channel. This result contrasts with i) DCPIB inhibition of native VRACs in lipid bilayers, where the inhibitor was applied to the cis-droplet (e.g., cytosolic channel orientation (Fig. 1K, ref PMID: 26824658), and ii) recent findings suggesting that membrane accumulation or permeation of DCPIB would be required for native VRAC inhibition (PMID: 41756460). The authors argue that DCPIB may access the channels from the outer constriction site (OCS), and one explanation could be a chimera artifact due to the homo-heptameric configuration of 8C-8AIL1^25 VRACs, which have a significantly wider and hydrophobic constriction (L105 residues) compared to native VRACs, which are dominated by positively charged R103 residues. Native VRACs composed of LRRC8A/C are highly selective for hydrophilic molecules, such as cyclic dinucleotides, whereas DCPIB is far from hydrophilic. Therefore, it's more likely that the drug accesses the channel from the lipidic membrane than the OCS, but the 8C-8AIL1^25 chimera could be an exception. To validate their findings and support their observations, I recommend that the authors test the effect of DCPIB in their 5-KO cells reconstituted with WT 8A+ 8C channels.

3. The section describing the 8C-8AIL1^25 M48K and M48R mutants, and especially the conclusions drawn from these observations, is somewhat unclear to me. The authors state that these mutants exhibit normal volume sensitivity; however, in Supplemental Fig. 1, their baseline outward and inward currents start around +/- 50 pA/pF at both +100 and -100 mV. In contrast, currents from the 8C-8AIL1^25 chimera start from ~0 pA/pF (Fig. 3F). Does this suggest that these mutant channels are pre-activated and that could account for their lack of DCPIB sensitivity?

4. Some of the 8C-8AIL1^25 chimera mutants (e.g., L136K and E6A) render the effect of DCPIB non-washable upon buffer exchange. It would be interesting to know whether these same mutations produce a similar effect in 8A-8C heteromers.

5. It would also be helpful to include a model in the concluding figure to visualize the hypothetical position of DCPIB within the channel. The authors discuss potential interactions of the drug with residues in EL1 and TM2, suggesting that its hydrophobic core may interact with M48 and Y126 at the vestibule, while its carboxylic group may reach L136. A structural representation indicating the relative size of the molecule and distances between these residues would greatly aid interpretation.

6. In the discussion section, there are many incorrect statements that should be taken care of: i) In the first page, last paragraph, last line, the M139L replacement experiment was done in the 8A-8C(EL1+TM2) chimera, not 8C, please correct. ii) First paragraph of Page 4, the authors propose that DCPIB inserts deeply into the pore or pore-lining inter-subunit cleft, positioning its carboxylate group within the conduction pathway where "it can directly engage charged residues such as L136K". None of the LRRC8 paralogs, except for the mutants the authors have tested, have a lysine at that position, but they all conserve leucine, making this statement irrelevant. Structural analysis will be required to support these hypothetical models, so I'd encourage the authors to soften their conclusions at the end of the discussion.

7. The text narrative in the result section, figure legends, and the selection of colors in some of the figures seem disorganized and require further work. For example, in Fig. 1, the first description focuses on panels C and I, then D and I, and so on, but 1A comes later in the text, and 1B is not mentioned. The same is true for Fig. 2B, which appears in the text after referencing Panel 2C. Many Figure panels are missing in the main text, e.g., Fig. 1B, 6A, 7B, 8B-D. Fig legend 9C appears before 9 B. Please reorganize. Many sections in the results limit their descriptions to the outcomes, and there is no overall observation. All these issues make the text and narrative hard to follow. It would be beneficial to add one sentence at the end of each section in the results summarizing the main findings of that section. The colors of the bars in Fig.2C are inconsistent with the chimeras/mutants shown in the rest of Figure 2.

Minor comments:

1. In the Introduction, 2nd page, line 3, include after (S1P): "in a"... nerve injury model.

2. Results section, Chimeric analysis of DCPIB-dependent LRRC8 channel inhibition:
a) The sentence "8A chimeras containing EL1 and TM2 from 8C, termed 8A-8C(IL1) and 8A-8C(TM2), respectively, ..." should likely read "8A-8C(EL1) and 8A-8C(TM2)."
b) In the final sentence of this section, "Taken together, these data suggest that hydrophobic packing between TM2 and TM2..." should be "between EL1 and TM2."

3. Fig 3F, the description of the 10 uM DCPIB application in the bath solution is missing. Please add to the description.

4. Is the 10 uM DCPIB application for channel activation in Fig. 5E done in a hypotonic bath solution? I guess it is, but it is not indicated in the text, figure legends, or methods section.

5. The Results section subtitle "Association between inactivation in DCPIB sensitivity" sounds unclear. It would likely be better phrased as "Association between inactivation and DCPIB sensitivity."

6. Fig. Leg. 1B, "blue" instead of "bule".

7. Use paralogs instead of orthologs when referring to other LRRC8 proteins than 8A (use at least 4 times in the text). Orthologs refer to the homolog proteins in different species.

Reviewer #2 (Comments to the Authors):

In this paper entitled, "State-dependent deep-pore inhibition of LRRC8 (VRAC) channels by DCPIB," the authors investigate the structural and mechanistic basis by which the small-molecule inhibitor DCPIB inhibits LRRC8 volume-regulated anion channels (VRACs). The rationale for this study stems from the widespread use of DCPIB as a canonical VRAC inhibitor despite incomplete understanding of its molecular mechanism of action, particularly in heteromeric LRRC8 channels. Using chimeric channel constructs, targeted mutagenesis, and whole-cell patch clamp electrophysiology in LRRC8-null cells, the authors systematically examine how specific extracellular and transmembrane domains regulate DCPIB sensitivity and channel gating behavior.

Using these approaches, the authors show that robust DCPIB inhibition requires coordinated contributions from extracellular loop 1 (EL1) and transmembrane domain 2 (TM2), which together form a functional module governing drug sensitivity. Within TM2, a single hydrophobic residue (M139/L137 position) strongly modulates DCPIB efficacy, suggesting that subtle changes in helix packing influence inhibitor engagement. Experiments using the homo-heptameric 8C-8A(IL125) chimera demonstrate that DCPIB binds from the extracellular side, possibly inhibits cooperatively, and enhances voltage-dependent inactivation, consistent with stabilization of an inactivated channel state. Additional mutational analyses across the outer constriction site, inter-subunit cleft, lipid-interacting residues, and N-terminal domain suggest that DCPIB penetrates deeply into the pore and

interacts with hydrophobic clefts to stabilize a non-conducting conformation. Together, these findings support a model in which DCPIB acts as a state-dependent inhibitor that couples pore occupancy to lipid gating and voltage-dependent inactivation mechanisms.
However, while the work provides compelling functional insights, a few relatively minor issues remain regarding methodology and inclusion of key controls that would improve the clarity and rigor of the manuscript prior to publication.

Specific Comments

1. Please include an example or description of the protocol used for quantifying voltage-dependent inactivation (VDI), both in the Methods section and as an explicit voltage protocol in Figures 3G-H. How long are the depolarizing test potentials used to generate these data?

2. Figure 2: Please include representative raw current traces plotted as current versus time, as well as representative I-V curves derived from the voltage ramp protocols that yielded these data.

3. Figure 3F: The finding that DCPIB inhibition of the 8C-8A(IL125) chimera does not occur from the intracellular side is very interesting and somewhat surprising, particularly given the lipophilic nature of DCPIB, which would be expected to partition into the plasma membrane and potentially access intracellular binding sites. Is this phenomenon unique to the 8C-8A(IL125) chimeric channel, or is it also observed for endogenous LRRC8 channel complexes in HCT cells studied in Figure 9 or other commonly used cell lines? Similarly, is this property observed in the 8A/C heteromer studied in Figure 8?

4. The shift in voltage-dependent inactivation shown in Figure 3H is also intriguing and represents a key finding with respect to the mechanism of DCPIB-mediated VRAC inhibition. This observation warrants further examination. In this experiment, 2 μM DCPIB is used-does a concentration-dependent shift in VDI occur? Under these experimental conditions, does DCPIB display use-dependent block, for example during repeated depolarizing steps to +60 mV with short interpulse intervals?

5. Figure 5B: These data are not explicitly referenced in the Results section when describing L136K current characteristics ("L136K currents exhibited normal volume sensitivity but abnormal voltage-dependent properties, including exaggerated VDI, loss of rectification, and time-dependent activation of inward current at hyperpolarized test potentials"). It would also be helpful to include representative raw traces for the WT 8C-8A(IL125) channel for comparison.

6. Figure 5D: The observation of incomplete washout is an interesting result. Please include representative raw traces from the ramp protocols and ideally show the full sequence of events: initial inhibition of hypotonically activated VRAC currents with DCPIB in both WT 8C-8A(IL125) and L136K mutants, followed by washout in hypotonic solution. As currently presented, the data appear to show only part of the experiment.

7. Figure 5C: The L136E mutant exhibited no inhibition by DCPIB at doses up to 3 μM. Why was testing limited to 3 μM? To more rigorously test this hypothesis, it would be helpful to extend the concentration range to 10 μM, as performed for other mutants, or potentially higher.

8. Figure 5E: Activation by DCPIB in this mutant is also a fascinating observation. Please include representative raw traces for this experiment. Additionally, it would be interesting to test analogous mutations in an 8A/C heteromer to determine whether similar activation phenomena are observed.

9. Figures 6 and 7: Several datasets in these figures are not explicitly referenced in the Results section (e.g., Figures 6A-B and 7B). Please ensure that all presented data are clearly described and interpreted in the text.

Reviewer #3 (Comments to the Authors):

The manuscript describes an electrophysiological analysis of inhibition of VRAC channels by the relatively high affinity compound DCPIB using heterologous expression combined with mutagenesis of various constructs, including homomeric LRRC8A, a chimera, and co-expressed LRRC8A and LRRC8C.

The question of the mechanism of inhibition by DCPIB has plagued the field for several years and indeed no clear picture has emerged so far.

The work definitely reports various interesting results regarding the effect various interventions on the effect of DCPIB. However, overall the manuscript is somehow a hodgepodge of various constructs and interpretations that unfortunately does not provide a coherent overall picture. For example effects of mutations in the "outer constriction site" are interpreted in terms of the structure of homomeric 8A with DCPIB bound, even though homomeric 8A seems to be insensitive to DCPIB - I don't understand how the authors interpret this.

A clear caveat is that many effects, in particular if they are small, could be indirect and not reflecting direct participation of the mutated regions in DCPIB binding. This appears to be the case in particular for the "fenestration" mutations.

A further point is that the "lipid gating hypothesis" is interpreted too much as a proven fact by the authors.

In general, the manuscript should be streamlined and more focused on a specific conclusion. Furthermore, it should be stressed that DCPIB is a highly unspecific compound that has effects on numerous other targets, including ion channels.

Minor comments:

For all figures it should be specified how currents before drug application were activated and for how long (i.e. hypotonicity, low ionic strength, constitutive). Regarding activation by hypotonicity / low ionic strength: was activation complete?

The sentence "Reconstitution of swelling-activated VRACs requires the expression of the essential 8A subunit along with at least one other paralog." needs a reference.

In the sentence "Gene-knockout studies have uncovered unexpected and potentially druggable roles of LRRC8/VRACs in metabolic, immune, and neurological disorders", what is a druggable role?
Regarding the sentence "Interestingly, however, the small-molecule VRAC inhibitor, DCPIB, improves glycemic control in T2D murine models by enhancing 8A/SWELL1 expression and insulin-PI3K-AKT signaling.", it is not acceptable to use DCPIB in mouse models because of the above mentioned myriads of effects of the drug on other known and probably many more unknown targets. Such studies should be cited in a more critical manner.

The sentence "These results indicate that synergistic interactions between EL1 and TM2 of 8C are needed for strong channel inhibition by DCPIB" is highly speculative. Effects could be indirect.

The "4 amino acids differing between the two orthologs (Fig. 1A)" should be highlighted in Fig. 1A.

The sentence "Taken together, these data suggest that hydrophobic packing between TM2 and TM2 influences channel sensitivity to DCPIB." is unclear.

Regarding the sentence "Cryo-EM and molecular docking studies have shown that DCPIB blocks the pore of homomeric 8A channels through a 'cork-in-bottle' mechanism of action" - what do the authors think about that?

Editor

1. JGP requires the use of appropriate statistical tests to establish the significance of your interventions, particularly for the data in Figures 1 and 2 but also in the dose response data in later figures.

We thank the editor for highlighting the need for clearly defined statistical analyses and apologize for the omission of this information in the original submission. In the revised manuscript, we have added a Statistical Analysis subsection to the Methods, as follows.

Data are presented as mean ± standard error of the mean (SEM), n represents the number of cells in patch clamp recordings. GraphPad Prism 11 software was used for all statistical analysis. Concentration response curves were generated by the fitting of four parameters Hill equation with constraints bottom and top to 0 and 100, respectively. V0.5 was determined by the fitting of Boltzmann equation. Statistical significance was determined by one-way analysis of variance (ANOVA) followed by Dunnett's multiple comparisons test for groups greater than two.

These revisions clarify that appropriate statistical methods have been applied consistently throughout the manuscript and are now explicitly described in the Methods and figure legends.

Reviewer 1

1. I will encourage the authors to present representative immunofluorescence images of fluorescent-tagged chimeras and mutants to ensure proper cell-surface expression (which will reflect proper channel folding, assembly, maturation, and trafficking).

We appreciate the reviewer's concern regarding potential variability in membrane expression of mutant and chimeric channels and agree that establishing functional expression is important. We considered this suggestion; however, we believe electrophysiology provides a more direct approach to this concern, as discussed below.

While GFP tagging can confirm general cellular localization, for our purposes it is not a reliable measure of relative membrane expression or density. Furthermore, and importantly, C-terminal GFP tagging has been shown to alter channel function, likely due to disruption of subunit assembly, conformational dynamics, or regulatory interactions within this multi-subunit complex. As such, GFP tagging does not necessarily report on the same population of channels that are functionally assessed electrophysiologically and may introduce confounding effects on channel behavior.

In contrast, whole-cell current density provides a direct, quantitative, and functionally relevant measure of membrane-expressed channels, as it reflects only channels that are properly folded, assembled, trafficked, and actively conducting at the cell surface. This approach inherently excludes non-functional or intracellular protein pools and allows normalization across cells by capacitance. Importantly, the primary conclusions of this study are based on qualitative changes in channel properties—such as voltage dependence, kinetics, and pharmacological responses, which are independent of absolute expression levels.

To further address the reviewer's concern, we have now included a new table 1 containing mean ± SEM current density (pA/pF) for all constructs used in this study, enabling direct comparison of functional membrane expression across chimeras and heteromers. Taken together, we believe that electrophysiological measurements provide a more rigorous and relevant assessment of channel expression than fluorescence-based approaches, and that our conclusions are not confounded by variability in membrane expression.

2. I'm intrigued by the author's findings that a 10 μM application of DCPIB in the pipette solution is insufficient to prevent swelling-induced channel activation (Fig 3F), which led them to conclude that the DCPIB binding site is accessible only from the extracellular side of the channel. To validate their findings and support their observations, I recommend that the authors test the effect of DCPIB in their 5-KO cells reconstituted with WT 8A+ 8C channels.

This is an excellent suggestion made by both Reviewers 1 and 2. We have now performed the requested experiments with WT 8A/8C channels in HCT-KO cells and native VRAC in WT HCT cells. Consistent with our findings using the IL25 chimera, inclusion of 30 μM DCPIB in the pipette solution did not prevent swelling-induced activation of 8A/8C or native VRAC channels. These results further support our conclusion that the DCPIB binding site is not accessible from the intracellular side and instead is reached from the extracellular face of the channel. These data are included in new Fig. S4.

3. The section describing the 8C-8AIL1^{25} M48K and M48R mutants, and especially the conclusions drawn from these observations, is somewhat unclear to me. The authors state that these mutants exhibit normal volume sensitivity; however, in Supplemental Fig. 1, their baseline outward and inward currents start around +/- 50 pA/pF at both +100 and -100 mV. In contrast, currents from the 8C-8AIL1^{25} chimera start from ~0 pA/pF (Fig. 3F). Does this suggest that these mutant channels are pre-activated and that could account for their lack of DCPIB sensitivity?

Thank you for commenting on this important detail. We have made the following change:

"Both mutants exhibited significantly higher baseline current but were further activated by cell swelling (Fig. S3), consistent with at least partially intact lipid gating, and exhibited outward rectification and pronounced VDI at depolarized potentials (Fig. 6B; Table 1)."

However, we do not believe that a pre-activated state can account for the loss of DCPIB sensitivity in these mutants because WT channels activated with hypotonic cell swelling are fully inhibitable by DCPIB.

4. Some of the 8C-8AIL1^{25} chimera mutants (e.g., L136K and E6A) render the effect of DCPIB non-washable upon buffer exchange. It would be interesting to know whether these same mutations produce a similar effect in 8A-8C heteromers.

We have performed new experiments to address this important point and found that, unlike washout of 8C-8AIL1^{25}-L136K and 8C-8AIL1^{25}-E6A, in which DCPIB was not reversible, washout of DCPIB from heteromeric 8A/8C channels containing these mutations occurred with the same extent and approximate timecourse of their wild type counterparts. We have added these data to a new Fig. S5 and include the following section to Discussion:

"The differential washout behavior of DCPIB between 8C-8A(IL1^{25}) and 8A/8C indicates that subunit composition governs not only apparent affinity but also the kinetics of drug dissociation. In 8C-8A(IL1^{25}), mutations that enhance DCPIB block (i.e., E6A or L136K) markedly reduce reversibility, consistent with stabilization of a drug-bound state with prolonged residence time. One potential explanation is that homomeric 8C-8A(IL1^{25}) assembly, derived predominantly from 8C, exhibits greater conformational flexibility, permitting induced-fit rearrangements that optimize interactions with DCPIB after binding. Such rearrangements may involve coordinated movements of pore-lining elements that effectively "tighten" around the ligand, increasing the energetic barrier for dissociation. In contrast, incorporation of 8A into heteromeric channels may impose structural constraints that favor

a more pre-organized binding site, thereby limiting induced-fit stabilization and promoting more complete washout.

Additionally, the homomeric organization of 8C-8A(IL1^{25}) may enable cooperative interactions among identical subunits, allowing symmetrical contributions to ligand coordination that are disrupted in the structurally heterogeneous 8A/8C assembly. This could further deepen the energy well of the DCPIB-bound state in 8C-8A(IL1^{25}) relative to heteromers. An alternative, but not mutually exclusive, possibility is that binding-induced conformational changes in 8C-8A(IL1^{25}) restrict solvent-accessible egress pathways, kinetically trapping DCPIB within the pore. Finally, stronger allosteric coupling between the DCPIB binding site and channel gating in 8C-8A(IL1^{25}) may stabilize a non-conducting conformation that further disfavors ligand dissociation. Together, these considerations support a model in which subunit-dependent differences in flexibility, cooperativity, and allosteric coupling shape the energy landscape of blocker binding, thereby determining both affinity and reversibility in LRRC8 channels."

5. It would also be helpful to include a model in the concluding figure to visualize the hypothetical position of DCPIB within the channel. The authors discuss potential interactions of the drug with residues in EL1 and TM2, suggesting that its hydrophobic core may interact with M48 and Y126 at the vestibule, while its carboxylic group may reach L136. A structural representation indicating the relative size of the molecule and distances between these residues would greatly aid interpretation.

Great suggestion! We have now added a new Fig. 10 and accompanying Legend that clarifies this point.

6. In the discussion section, there are many incorrect statements that should be taken care of: i) In the first page, last paragraph, last line, the M139L replacement experiment was done in the 8A-8C(EL1+TM2) chimera, not 8C, please correct.

We apologize for this oversight and have corrected the sentence in the revised manuscript.

ii) First paragraph of Page 4, the authors propose that DCPIB inserts deeply into the pore or pore-lining inter-subunit cleft, positioning its carboxylate group within the conduction pathway where "it can directly engage charged residues such as L136K". None of the LRRC8 paralogs, except for the mutants the authors have tested, have a lysine at that position, but they all conserve leucine, making this statement irrelevant. Structural analysis will be required to support these hypothetical models, so I'd encourage the authors to soften their conclusions at the end of the discussion.

We agree with this comment and have softened the conclusions in this Discussion paragraph, as follows.

"The contrasting effects of charged substitutions at L136 in 8C-8A(IL1^{25}) provide functional evidence that DCPIB is influenced by electrostatic determinants located well inside the OCS."

"These opposing effects suggest that DCPIB experiences the electrostatic environment of residues deep within the pore."

"Distinguishing between direct deep-pore penetration of the blocker and long-range electrostatic modulation of OCS binding will require structural and computational studies capable of resolving the position of DCPIB within the pore and quantifying electrostatic coupling between these sites."

7. The text narrative in the result section, figure legends, and the selection of colors in some of the figures seem disorganized and require further work. For example, in Fig. 1, the first description focuses on panels C and I, then D and I, and so on, but 1A comes later in the text, and 1B is not mentioned. The same is true for Fig. 2B, which appears in the text after referencing Panel 2C. Many Figure panels are missing in the main text, e.g., Fig. 1B, 6A, 7B, 8B-D. Fig legend 9C appears before 9 B. The colors of the bars in Fig. 2C are inconsistent with the chimeras/mutants shown in the rest of Figure 2. Please reorganize.

We have corrected the figures and associated text as requested.

8. Many sections in the results limit their descriptions to the outcomes, and there is no overall observation. All these issues make the text and narrative hard to follow. It would be beneficial to add one sentence at the end of each section in the results summarizing the main findings of that section.

We completely agree with the Editor and this Reviewer that the manuscript will be improved by including a unifying narrative to help the reader understand the order and logic of experiments. We have therefore added a new paragraph in the Results section and descriptive section titles to help the reader understand the progression, as follows.

“Conceptual framework for DCPIB inhibition
Previous structural and docking studies suggest that DCPIB inhibits LRRC8 channels through a pore-blocking mechanism involving interactions at the extracellular vestibule and within inter-subunit clefts. These observations led us to hypothesize a stepwise mechanism in which DCPIB, when applied extracellularly, initially engages residues in the EL1 domain near the outer constriction site (OCS), then partitions into hydrophobic inter-subunit clefts (“fenestrae”), and subsequently diffuses along the membrane-facing interface toward deeper regions of the pore. In this model, the hydrophobic tail of DCPIB facilitates its stabilization within lipid-exposed clefts, enabling access to transmembrane residues and ultimately to deeper pore-lining positions, where the drug may stabilize a blocked and/or inactivated state.”

Section headings are now:

- *EL1 - OCS (first contact)*
- *EL1 - inter-subunit cleft (entry into fenestrae)*
- *TM2 mutagenesis (downward progression along lipid interface)*
- *L136 (transition to pore engagement)*
- *“Lipid gate” mutagenesis*
- *NTD mutagenesis (deepest site/endpoint)*

Minor comments:

1. In the Introduction, 2nd page, line 3, include after (S1P): "in a"... nerve injury model.

Done

2. Results section, Chimeric analysis of DCPIB-dependent LRRC8 channel inhibition:
a) The sentence "8A chimeras containing EL1 and TM2 from 8C, termed 8A-8C(IL1) and 8A-8C(TM2), respectively, ..." should likely read "8A-8C(EL1) and 8A-8C(TM2)."

Thank you for catching this mistake. We have corrected it as requested.

b) In the final sentence of this section, "Taken together, these data suggest that hydrophobic packing between TM2 and TM2..." should be "between EL1 and TM2."

Thank you. Done.

3. Fig 3F, the description of the 10 uM DCPIB application in the bath solution is missing. Please add to the description.

Done

4. Is the 10 uM DCPIB application for channel activation in Fig. 5E done in a hypotonic bath solution? I guess it is, but it is not indicated in the text, figure legends, or methods section.

We agree that this was not made clear and have made the following changes:

(Methods): "DCPIB inhibition experiments were performed following steady-state activation of LRRC8 currents by hypotonic cell swelling."

(Results): "Unexpectedly, however, 10 µM DCPIB led to a striking additional activation of swelling-induced 8C-8A(IL1[25])-L136E currents (Fig. 5E)."

(Figure Legend): "(E) Time-course of additional DCPIB-dependent activation of swelling-induced L136E mutant (n = 5)."

5. The Results section subtitle "Association between inactivation in DCPIB sensitivity" sounds unclear. It would likely be better phrased as "Association between inactivation and DCPIB sensitivity."

Done

6. Fig. Leg. 1B, "blue" instead of "bule".

Done

7. Use paralogs instead of orthologs when referring to other LRRC8 proteins than 8A (use at least 4 times in the text). Orthologs refer to the homolog proteins in different species.

Apologies for this oversight. We have made the correction throughout the manuscript.

Reviewer #2

1. Please include an example or description of the protocol used for quantifying voltage-dependent inactivation (VDI), both in the Methods section and as an explicit voltage protocol in Figures 3G-H.

We have included the following sentence in the Methods section and added a voltage clamp protocol to Fig 3:

"Voltage-dependent inactivation (VDI) was estimated from "tail currents" recorded at -80 mV following each voltage step between -120 mV and +120 mV and normalized to that at 0 mV, where tail-current amplitude was greatest."

How long are the depolarizing test potentials used to generate these data?

Voltage steps were 2 sec, as described in the Methods section:

"Current-to-voltage (I-V) relationships were established by stepping for 0.5 sec to -80 mV followed by 2-sec steps between -120 mV and +120 mV in 20 mV increments."

2. Figure 2: Please include representative raw current traces plotted as current versus time, as well as representative I-V curves derived from the voltage ramp protocols that yielded these data.

We include representative IV traces recorded in control buffer and following steady state inhibition by DCPIB in a new Fig. S1.

3. Figure 3F: The finding that DCPIB inhibition of the 8C-8A($IL1^{25}$) chimera does not occur from the intracellular side is very interesting and somewhat surprising, particularly given the lipophilic nature of DCPIB, which would be expected to partition into the plasma membrane and potentially access intracellular binding sites. Similarly, is this property observed in the 8A/C heteromer studied in Figure 8?

We have performed the requested experiments with WT 8A/8C channels and included the data in a new Fig. S5. Consistent with our findings using the $IL1^{25}$ chimera, inclusion of 30 μM DCPIB in the pipette solution did not prevent swelling-induced activation of 8A/8C heteromeric channels or subsequent inhibition with bath applied DCPIB. These results further support our conclusion that the DCPIB binding site is not accessible from the intracellular side and instead is reached from the extracellular face of the channel.

Is this phenomenon unique to the 8C-8A($IL1^{25}$) chimeric channel, or is it also observed for endogenous LRRC8 channel complexes in HCT cells studied in Figure 9 or other commonly used cell lines?

We performed this experiment on native VRAC in HCT cells and found that intracellular dialysis of 30 uM DCPIB does not inhibit VRAC. These data are included in Fig. S5.

4. The shift in voltage-dependent inactivation shown in Figure 3H is also intriguing and represents a key finding with respect to the mechanism of DCPIB-mediated VRAC inhibition. This observation warrants further examination. In this experiment, 2 μM DCPIB is used-does a concentration-dependent shift in VDI occur?

While this is a great suggestion, unfortunately the small current amplitude and tail current variability with DCPIB inhibition prevent a statistically meaningful analysis of the dose-responsiveness.

Under these experimental conditions, does DCPIB display use-dependent block, for example during repeated depolarizing steps to +60 mV with short interpulse intervals?

Interesting idea! We performed these experiments and found no evidence of use dependence. These data are included in a new Fig. S6.

5. Figure 5B: These data are not explicitly referenced in the Results section when describing L136K current characteristics ("L136K currents exhibited normal volume sensitivity but abnormal voltage-dependent properties, including exaggerated VDI, loss of rectification, and time-dependent activation of inward current at hyperpolarized test potentials").

We now include a new Table 1 containing the electrophysiological properties of all 8C-8A($IL1^{25}$) and 8A/8C constructs used in our study.

It would also be helpful to include representative raw traces for the WT 8C-8A($IL1^{25}$) channel for comparison.

While we agree that including WT 8C-8A($IL1^{25}$) current traces would be helpful for comparison, the same argument could be made for every figure showing mutant data. For this reason, and because Fig. 5B is already “busy”, we prefer not to include WT representative trace and will refer the reader back to Fig. 3.

6. Figure 5D: The observation of incomplete washout is an interesting result. Please include representative raw traces from the ramp protocols and ideally show the full sequence of events: initial inhibition of hypotonically activated VRAC currents with DCPIB in both WT 8C-8A(IL125) and L136K mutants, followed by washout in hypotonic solution. As currently presented, the data appear to show only part of the experiment.

We now show DCPIB wash-in, steady state inhibition, and washout in Fig. 5D and include representative IV traces in a new Fig. S4.

7. Figure 5C: The L136E mutant exhibited no inhibition by DCPIB at doses up to 3 μM. Why was testing limited to 3 μM? To more rigorously test this hypothesis, it would be helpful to extend the concentration range to 10 μM, as performed for other mutants, or potentially higher.

We separated out the lower-dose CRC (up to 3 μM) from the 10 μM dose because there is no effect of lower doses of DCPIB up to 3 μM (Fig. 5C). We include activation data from the 10 μM dose in Fig. 5E because including it in the Fig. 5C CRC would impose a large “negative activation” in that curve.

8. Figure 5E: Activation by DCPIB in this mutant is also a fascinating observation. Please include representative raw traces for this experiment.

These data are now shown in Fig. S2B.

Additionally, it would be interesting to test analogous mutations in an 8A/C heteromer to determine whether similar activation phenomena are observed.

Thank you for this suggestion! We performed these experiments and found, remarkably, that 8A-L134E/8C-L136E channels not only retain sensitivity to DCPIB, unlike their IL25 counterparts, but

they actually exhibit slightly higher sensitivity. We include new text in the Discussion discussing this interesting observation.

"In contrast, substitution with glutamate (L136E) abolished low-dose DCPIB inhibition in the chimera but slightly enhanced DCPIB in 8A/8C. One explanation is that placement of a charged residue at position 136 promotes reorientation of TM2. Because glutamate is shorter and less conformationally flexible than lysine, its ability to influence pore electrostatics may depend on a larger structural rearrangement that repositions the side chain toward the pore. Such rearrangements may be favored in the more conformationally dynamic 8C-8A(IL1[25]) but constrained in 8A-containing channels. Under this model, lysine can enhance DCPIB block in both channel backgrounds because its longer, more flexible side chain can extend toward the pore without requiring substantial backbone movement, whereas glutamate exerts a strong effect only in channels that permit TM2 repacking or rotation. These findings together with observations that destabilizing mutations at L137/M139 enhance DCPIB sensitivity (discussed above) support the idea that subunit composition influences not only DCPIB sensitivity but also the structural consequences of mutations within the TM2 fenestral region, revealing a coupling between channel dynamics and the electrostatic determinants of blocker action. Cryo-EM and computational studies will be required to rigorously test this hypothesis."

9. Figures 6 and 7: Several datasets in these figures are not explicitly referenced in the Results section (e.g., Figures 6A-B and 7B). Please ensure that all presented data are clearly described and interpreted in the text.

We apologize for this oversight and now reference all data panels in the revised manuscript.

Reviewer #3

1. The effects of mutations in the "outer constriction site" are interpreted in terms of the structure of homomeric 8A with DCPIB bound, even though homomeric 8A seems to be insensitive to DCPIB - I don't understand how the authors interpret this.

We now critically discuss the cork-in-the-bottle model in the opening paragraph of the Discussion section.

2. A clear caveat is that many effects, in particular if they are small, could be indirect and not reflecting direct participation of the mutated regions in DCPIB binding. This appears to be the case in particular for the "fenestration" mutations.

We completely agree that some effects of mutations on DCPIB pharmacology may be indirect and state this explicitly in the revised Discussion section, as follows.

"A limitation of the present study is that changes in DCPIB sensitivity produced by point mutations cannot be interpreted exclusively as evidence for direct drug–residue interactions. Many of the residues examined reside within tightly coupled structural modules that govern pore architecture, inter-subunit packing, lipid occupancy, and gating transitions. As a result, mutations may indirectly alter DCPIB pharmacology by perturbing channel conformation, dynamics, or state equilibria rather than by disrupting specific binding contacts. We have sought to mitigate these limitations by combining mutagenesis with structural context, bidirectional electrostatic perturbations (e.g., L136K vs. L136E), and analysis of correlated functional effects, such as changes in reversibility, Hill slope, and voltage dependence. Nevertheless, definitive assignment of direct binding interactions will

require complementary approaches, including structural determination of drug-bound states and systematic chemical modification of DCPIB. Accordingly, the mechanistic model proposed here should be viewed as an experimentally constrained framework that integrates both direct and indirect contributions of multiple channel domains to DCPIB inhibition."

3. A further point is that the "lipid gating hypothesis" is interpreted too much as a proven fact by the authors.

We agree and have softened this interpretation throughout the manuscript, including this new sentence in the Discussion section:

"These observations have led to the so-called "lipid gating" hypothesis, where channel activation is caused by "de-lipidation" of the pore."

4. In general, the manuscript should be streamlined and more focused on a specific conclusion..

We agree that the original manuscript lacked clear organization that conveys our experimental approach. As noted above in our response to Reviewer 1, we have added a new paragraph and section titles to provide better organization and clarity for the reader. We hope this improved organization style is acceptable to both Reviewers.

Furthermore, it should be stressed that DCPIB is a highly unspecific compound that has effects on numerous other targets, including ion channels.

We have added the following sentence to the Discussion section:

"Given that DCPIB is widely known to be a promiscuous VRAC blocker with numerous off-targets, there is a critical need for developing new classes of more potent and selective inhibitors."

5. For all figures it should be specified how currents before drug application were activated and for how long (i.e. hypotonicity, low ionic strength, constitutive). Regarding activation by hypotonicity / low ionic strength: was activation complete?

We include the following sentence in the Methods section:

"Cells were swollen by exposure to hypotonic bath solution (250 mOsm) made by removal of sucrose. DCPIB inhibition experiments were performed following steady-state activation of LRRC8 currents by hypotonic cell swelling. Experiments were performed at room temperature."

6. The sentence "Reconstitution of swelling-activated VRACs requires the expression of the essential 8A subunit along with at least one other paralog." needs a reference.

We have added the reference.

7. In the sentence "Gene-knockout studies have uncovered unexpected and potentially druggable roles of LRRC8/VRACs in metabolic, immune, and neurological disorders", what is a druggable role?

We apologize for this use of jargon, and have changed this sentence, as follows:

"Gene-knockout studies have uncovered unexpected roles of LRRC8/VRACs in metabolic, immune, and neurological disorders that may represent new therapeutic opportunities."

8. Regarding the sentence "Interestingly, however, the small-molecule VRAC inhibitor, DCPIB, improves glycemic control in T2D murine models by enhancing 8A/SWELL1 expression and insulin-PI3K-AKT signaling.", it is not acceptable to use DCPIB in mouse models because of the above mentioned myriads of effects of the drug on other known and probably many more unknown targets. Such studies should be cited in a more critical manner.

We agree and have edited this section as follows:

"While this observation supports the idea that inhibition of VRAC might offer a new therapeutic approach to improving blood glucose homeostasis in metabolic syndrome, critical validation studies will require the development of more potent and selective inhibitors. Rajan Sah and colleagues recently reported cryo-EM structure-guided medicinal chemistry efforts to identify DCPIB analogs with improved potency toward VRAC and in vivo efficacy[17]."

9. The sentence "These results indicate that synergistic interactions between EL1 and TM2 of 8C are needed for strong channel inhibition by DCPIB" is highly speculative. Effects could be indirect.

We agree and have softened this statement, as follows:

"These results are consistent with the idea that cooperative interactions between EL1 and TM2 of 8C are needed for strong channel inhibition by DCPIB."

Additionally, state in the Discussion section that the effects of mutations on DCPIB could be indirect.

10. The "4 amino acids differing between the two orthologs (Fig. 1A)" should be highlighted in Fig. 1A.

We have highlighted these residues as requested.

11. The sentence "Taken together, these data suggest that hydrophobic packing between TM2 and TM2 influences channel sensitivity to DCPIB." is unclear.

We apologize for the typo and have changed this to:

"Taken together, these data are consistent with the notion that hydrophobic packing and associated conformation of TM2 influences channel sensitivity to DCPIB."

12. Regarding the sentence "Cryo-EM and molecular docking studies have shown that DCPIB blocks the pore of homomeric 8A channels through a 'cork-in-bottle' mechanism of action" - what do the authors think about that?

We thank the reviewer for the opportunity to address this proposed mechanism more directly. In response, we have added two new paragraphs to the Discussion that explicitly evaluate this model in the context of our new data, as follows:

"The proposed cork-in-the-bottle mechanism for DCPIB inhibition, based on cryo-EM structures and computational docking of 8A homomers, proposes that the drug occludes the

extracellular pore via interactions at the OCS near R103 while its hydrophobic tail partitions into membrane-facing fenestrations. While this model provides an important structural snapshot, our results indicate that it represents an early stage of a more dynamic inhibitory process. We considered a stepwise mechanism in which DCPIB first engages EL1 residues near the outer constriction, then partitions into inter-subunit fenestrations, and subsequently progresses along membrane-facing interfaces toward deeper regions of the pore. Our functional data supports this sequential model but further shows that effective inhibition requires coordinated interactions extending beyond the OCS. Although R103 is critical for inhibition in LRRC8A homomers, consistent with an initial binding step, its dispensability in 8A/8C heteromers indicates that this interaction is not required and therefore cannot fully account for channel block, arguing against a purely static extracellular plug mechanism.

We therefore propose a dynamic, state-dependent mechanism in which DCPIB binds extracellularly but undergoes sequential repositioning within the channel, ultimately accessing deeper pore-lining regions where it stabilizes an inactivated conformation. The cooperative nature of inhibition and its enhancement of VDI support stabilization of a specific functional state rather than simple pore occlusion. In this context, the cryo-EM structure likely represents a metastable, pre-inhibitory binding pose rather than the final inhibitory configuration. Thus, while the cork-in-the-bottle model captures an important initial interaction at the outer pore, it does not account for the depth, cooperativity, and state dependence of inhibition revealed here."

Dr. Jerod Denton
Vanderbilt University Medical Center
Anesthesiology
T4208 MCN
1161 21st Avenue South
Nashville, TN 37221

Re: 202614024R1

Dear Dr. Denton,

I am pleased to let you know that your manuscript, titled "State-Dependent Inhibition of LRRC8 Volume-Regulated Anion Channels by DCPIB" is scientifically acceptable for publication in Journal of General Physiology. The remaining comments from the reviewers should be straightforward to adress. Please also include P-values for the measurements shown in the bar graphs in Figures 1 and 2. Formal acceptance will follow when it is modified in accordance with the referees' remarks, the P-values are included, and our editorial policies are addressed.

Please note items that need attention are listed at the bottom of this email (under 'manuscript formatting checklist'). Please also be sure to include a letter addressing the reviewers' comments point-by-point (if applicable) and a copy of the text with alterations highlighted (boldfaced or underlined). Your manuscript should be a double-spaced MS Word file and include editable tables, if appropriate.

Lastly, JGP requires a data availability statement for all research article submissions. These statements will be published in the article directly above the Acknowledgments. The statement should address all data underlying the research presented in the manuscript. Please visit the JGP instructions for authors for guidelines and examples of statements at https://rupress.org/jgp/pages/editorial-policies#data-availability-statement.

Please submit your final files via this link:
Link Not Available

Thank you for choosing to publish your research in JGP and please feel free to contact me with any questions.

Sincerely,

Joseph A. Mindell, M.D., Ph.D.
On behalf of Journal of General Physiology

Journal of General Physiology's mission is to publish mechanistic and quantitative molecular and cellular physiology of the highest quality; to provide a best in class author experience; and to nurture future generations of independent researchers.

Manuscript formatting checklist:
- MS Word document of text needed (including editable tables)
- MS Word document of supplemental text needed, if applicable (including figure legends and editable tables)
- Brief Statement describing supplementary information needed, if applicable (in subsection at end of Materials & Methods)
- Reference should not be numbered and should follow these guidelines: https://rupress.org/jgp/pages/reference-guidelines
- Please include a data availability statement preceding the Acknowledgments section. Please see https://rupress.org/jgp/pages/editorial-policies#data-availability-statement
- Figures created at sufficient resolution and in acceptable format (including supplemental if applicable). If working in Illustrator, we prefer .ai or .eps file format. If working in Photoshop please use 600dpi/1000dpi .tiff or .psd file format. Minimum resolution at estimated print size: Minimum resolution for all figures is 600 dpi. For figures that contain both photographs and line art or text, 600 dpi is highly recommended. Figures containing only black and white elements (line art, no color, and no gray) should be 1,000 dpi. Maximum figure size is 7 in wide x 9 in high (17.5 x 22.8 cm) at the correct resolution. https://jgp.rupress.org/fig-vid-guidelines
- Supplemental figures, if any, conforming to same guidelines as manuscript figures (noted above)
- If images resemble one from a prior publications, the author must seek permissions (to reproduce or adapt) from the original publisher. [You can resubmit your paper while waiting to hear back from the original publisher but please keep us updated]
- All authors must complete a disclosure form prior to acceptance. A link to complete the form has been sent to all coauthors. Please provide the editorial office with updated email addresses if necessary

Reviewer #1 (Comments to the Authors):

I appreciate the authors for addressing most of my comments. I acknowledge that the paper has significantly improved following the revision. I have a minor comment and some thoughts to share with the authors, which I leave to their discretion to incorporate into the final version of the manuscript, should they find them useful.

Minor Comment:
1. Line 192: Please correct I33 to I133 in L131/I33, as there is a "1" missing.

Additional general thoughts to consider (if necessary):
- Proposed Model of DCPIB Inhibition: The authors propose that DCPIB accesses the channel via the Outer Constriction Site (OCS), mediated by electrostatic interactions between R103 residue in LRRC8A and the carboxylate group of DCPIB (new Figure 10), supporting the current Cryo-EM model as a primary step in the deep-pore engagement mechanism of inhibition. However, this interaction does not occur in the 8C-8A-IL1-25 chimera, which has a leucine residue at the homologous position and remains inhibited by DCPIB. To support their model, the authors suggest that K51 in LRRC8C may account for this interaction in the absence of R103, as R103 is neither required nor sufficient to explain DCPIB inhibition. This assumption was raised in the recent paper by the Estevez group (PMID: 42348849), in which the R103F mutant was introduced into LRRC8A in a heteromeric configuration of 8A/8E VRACs; however, there is no direct evidence that this interaction between the DCPIB carboxylate and the K51 in the 8C-8A-IL1-25 chimera occurs. The authors of that study indicate that mutating the K51 residue of LRRC8A renders the channel nonfunctional when R103 is absent. Therefore, the authors of this study based their conclusion on speculation rather than established evidence. In my opinion, I disagree with the authors' model suggesting that DCPIB may access the pore of VRACs through the OCS. I would speculate that access depends on DCPIB's interaction with the outer leaflet of the plasma membrane (not the electronegative inner leaflet side, as demonstrated by the authors). The selectivity filter of native VRACs, primarily composed of LRRC8A, may favor the attraction of hydrophilic rather than lipophilic small molecules such as DCPIB. While the amphipathic nature of DCPIB -due to its carboxylate group- might enable electrostatic interactions with positively charged R or K residues near the OCS, its high lipophilicity may create an energetic barrier that prevents DCPIB from approaching such a hydrophilic environment. Thus, the structure of the plasma membrane may facilitate DCPIB insertion, thereby enabling access to the inner sites of VRACs through intersubunit gaps. Therefore, one might speculate that DCPIB accesses VRACs via the plasma membrane rather than the OCS, as proposed by the authors, the current Cryo-EM model, and the aforementioned manuscript by the Estevez group. Deep-pore inhibition might still occur, as elegantly proposed by the authors, but DCPIB's access may not be directly through the OCS. Therefore, while the proposed model of DCPIB inhibition is interesting, it remains open to debate.

Physiological Implications of the Study: Although the current model proposes an alternative mechanism by which DCPIB inhibits VRACs, its implications lack physiological relevance. There are currently two preprint manuscripts that strongly suggest a lack of VRAC inhibition by DCPIB in the presence of albumin, a situation that occurs under physiological conditions (PMID: 41756460, PMID: 42523362). Therefore, while the model proposed by the authors may be of some interest for explaining an electrophysiological phenomenon that occurs artificially in the absence of albumin, it will not apply in physiological settings, thereby significantly diminishing its relevance.

Reviewer #2 (Comments to the Authors):

The authors have responded to appropriately to all concerns except for a few additional items below:

1. Thank you for studying the effect of internal DCPIB on both 8A/C channels and endogenous VRAC currents. As this effect is interesting and impactful it was important to show that this is relevant to an LRRC8 channel that exists in nature, as opposed to the 8C-8A(IL125) chimera channel. For this reason, I feel that this data is best placed among the main figures and not in the supplement.

2. OK I understand the rationale that testing 10 uM DCPIB on the L136E mutant would potentially cause further activation to hypotonically activated VRAC currents. Nonetheless, it would be interesting to show that if the data was available.

3. Thank you for providing raw IV traces for the L136E mutant showing massive VRAC activation - up to 16 nA! and this is entirely reversible with DCPIB washout. That current amplitude is nearly saturation for a standard Axon whole-cell patch-clamp amplifier, and it still retains some outward rectification. This is not a subtle result! Based on the importance of this result it would benefit from also being placed in the main figures.

4. Thank you for testing the 8A-L134E/8C-L136E as this was a bit of work. This result should be included in the manuscript instead of described in the discussion alone, as the DCPIB activation effect is clearly a feature of the 8C-8A(IL125) chimera channel as opposed to an 8A/C channel with the same mutation. This should be a clearly described result, as it is a very important one.

Reviewer #3 (Comments to the Authors):
The authors have adequately addressed my concerns

Editor

Please also include P-values for the measurements shown in the bar graphs in Figures 1 and 2.

We have added the P-values to the figures and legends.

Reviewer #1:

Minor Comment:
1. Line 192: Please correct I33 to I133 in L131/I33, as there is a "1" missing.

We have corrected this typo.

Additional general thoughts to consider (if necessary):
- Proposed Model of DCPIB Inhibition: The authors propose that DCPIB accesses the channel via the Outer Constriction Site (OCS), mediated by electrostatic interactions between R103 residue in LRRC8A and the carboxylate group of DCPIB (new Figure 10), supporting the current Cryo-EM model as a primary step in the deep-pore engagement mechanism of inhibition. However, this interaction does not occur in the 8C-8A-IL1-25 chimera, which has a leucine residue at the homologous position and remains inhibited by DCPIB. To support their model, the authors suggest that K51 in LRRC8C may account for this interaction in the absence of R103, as R103 is neither required nor sufficient to explain DCPIB inhibition. This assumption was raised in the recent paper by the Estevez group (PMID: 42348849), in which the R103F mutant was introduced into LRRC8A in a heteromeric configuration of 8A/8E VRACs; however, there is no direct evidence that this interaction between the DCPIB carboxylate and the K51 in the 8C-8A-IL1-25 chimera occurs. The authors of that study indicate that mutating the K51 residue of LRRC8A renders the channel nonfunctional when R103 is absent. Therefore, the authors of this study based their conclusion on speculation rather than established evidence. In my opinion, I disagree with the authors' model suggesting that DCPIB may access the pore of VRACs through the OCS. I would speculate that access depends on DCPIB's interaction with the outer leaflet of the plasma membrane (not the electronegative inner leaflet side, as demonstrated by the authors). The selectivity filter of native VRACs, primarily composed of LRRC8A, may favor the attraction of hydrophilic rather than lipophilic small molecules such as DCPIB. While the amphipathic nature of DCPIB -due to its carboxylate group- might enable electrostatic interactions with positively charged R or K residues near the OCS, its high lipophilicity may create an energetic barrier that prevents DCPIB from approaching such a hydrophilic environment. Thus, the structure of the plasma membrane may facilitate DCPIB insertion, thereby enabling access to the inner sites of VRACs through intersubunit gaps. Therefore, one might speculate that DCPIB accesses VRACs via the plasma membrane rather than the OCS, as proposed by the authors, the current Cryo-EM model, and the aforementioned manuscript by the Estevez group. Deep-pore inhibition might still occur, as elegantly proposed by the authors, but DCPIB's access may not be directly through the OCS. Therefore, while the proposed model of DCPIB inhibition is interesting, it remains open to debate.

Thank you for this comment. We have added the following paragraph to the end of the Discussion to address this point:

"Alternatively, DCPIB may access its deep pore binding site through a membrane-associated route rather than by entering directly through the aqueous vestibule at the OCS. Because DCPIB is highly lipophilic, partitioning into the outer leaflet of the plasma membrane could facilitate its approach to the channel through intersubunit gaps, with subsequent engagement of pore-lining residues responsible for inhibition. Such a mechanism is not mutually exclusive with the structural model inferred from cryo-EM studies, as electrostatic interactions involving residues near the OCS could still contribute to drug stabilization or positioning after entry. Although our findings are consistent with deep-pore inhibition by DCPIB, they do not definitively establish the route by which the inhibitor reaches its binding site, and alternative membrane-facilitated access pathways warrant future investigation."

Reviewer #2:

1. Thank you for studying the effect of internal DCPIB on both 8A/C channels and endogenous VRAC currents. As this effect is interesting and impactful it was important to show that this is relevant to an LRRC8 channel that exists in nature, as opposed to the 8C-8A(IL125) chimera channel. For this reason, I feel that this data is best placed among the main figures and not in the supplement.

We fully agree that these are important data that indicate that this is not a unique property of the IL25 chimera, however because these are negative data and would add additional panels to already busy figures, we prefer to keep the data as supplemental figures. However, we have added the following sentences to the Results section immediately after the IL25 data (Fig. 3) to emphasize this important detail. We hope that Reviewer 2 finds this acceptable.

"This is not a unique property of 8C-8A(IL1^{25}), as intracellular delivery of 30 µM DCPIB did not prevent swelling-induced activation of heteromeric 8A/8C channels or native VRAC expressed in wild type HCT cells (Fig. S2)."

2. OK I understand the rationale that testing 10 uM DCPIB on the L136E mutant would potentially cause further activation to hypotonically activated VRAC currents. Nonetheless, it would be interesting to show that if the data was available.

These data are included in Fig. 5E.

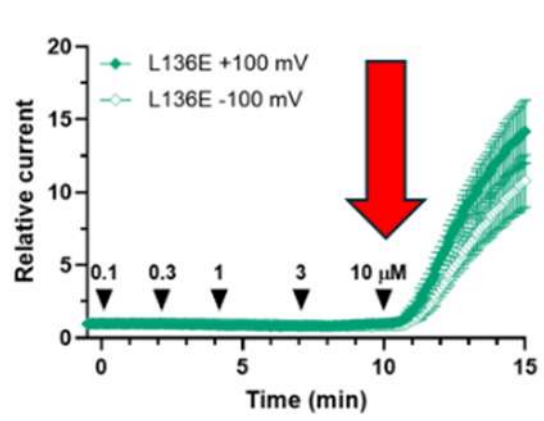

3. Thank you for providing raw IV traces for the L136E mutant showing massive VRAC activation - up to 16 nA! and this is entirely reversible with DCPIB washout. That current amplitude is nearly saturation for a standard Axon whole-cell patch-clamp amplifier, and it still retains some outward rectification. This is not a subtle result! Based on the importance of this result it would benefit from also being placed in the main figures.

We agree that these are striking effects and something we are deeply interested in exploring in future studies. However, we strongly prefer to keep the L136E representative IVs with the L136K and E6A data in Fig. S3.

4. Thank you for testing the 8A-L134E/8C-L136E as this was a bit of work. This result should be included in the manuscript instead of described in the discussion alone, as the DCPIB activation effect is clearly a feature of the 8C-8A(IL125) chimera channel as opposed to an 8A/C channel with the same mutation. This should be a clearly described result, as it is a very important one.

We included these data in the Results section (see below), Table 2 and Fig.8.

“Interestingly, whereas the L136E mutation eliminated DCPIB block of 8C-8A(IL1^{25}) (Fig. 5C; Table 2), the corresponding 8A-L134E/8C-L136E mutation led to a small but significant enhancement of DCPIB sensitivity (Fig. 8D; Table 2).”
